# Mantle melting and lithospheric structure beneath eastern Australia’s Cenozoic volcanoes from 3D magnetotellurics

**DOI:** 10.1038/s41598-026-44483-8

**Published:** 2026-03-19

**Authors:** Relly Margiono, Graham Heinson

**Affiliations:** 1 Department of Earth Sciences, Adelaide University, Adelaide, SA 5005 Australia; 2 Department of Geophysics, Indonesia State College of Meteorology, Climatology, and Geophysics, Tangerang, Indonesia

**Keywords:** Magnetotelluric, Volcano, Australia, Resistivity, Cenozoic, Planetary science, Solid Earth sciences

## Abstract

One of the most remarkable features of the eastern Australian continent are Cenozoic age (65 Ma to present) volcanoes over 3,000 km span north-south, with no significant age progression, even though the continent has moved at a rate of up to 75 km/Ma in an NNE direction over much of this time. Three theorems have been advanced to explain age-independent volcanism: (1) decompression melting from the transition zone due to volatile content from subducted slab stagnation; (2) edge-driven convection at the margins of steps in lithospheric thickness that drive upwelling from volatile-rich mantle reservoirs; and (3) melting of low-viscosity pockets of sub-lithospheric mantle due to asthenospheric shear. In this paper we have undertaken a 3D inversion of magnetotelluric (MT) sites across the Tasmanide accretionary orogens in eastern Australia (~ 800 locations) to define the broad-scale resistivity of the lithosphere and asthenosphere. The upper mantle directly beneath the age-independent Cenozoic volcanoes has anomalously low-resistivity (~ 100 Ohm.m) below 125 km depth, compatible with dry lherzolite-harzburgite-wehrlite compositions at temperatures of ~ 1,400 °C. The resistivity model suggests that inland from the volcanos, there is a step-like increase in lithospheric thickness. In the lower crust, beneath volcanic centres a resistivity of ~ 50 Ohm.m is consistent with a hydrated clinopyroxene-orthopyroxene-plagioclase composition at ~ 900 °C. No additional conduction mechanisms (such as graphite, sulphides or partial melt) are required. Results suggest that decompression melting from the transition zone raises the geotherm to adiabatic below 125 km and efficiently removes volatiles to the crust over a wide area (hundreds of kilometres) of subducted slab. Edge-driven convention may also occur at the step in the lithosphere thickness, particularly for the New Volcanic Zone and the leucite Cosgrove Track volcanoes. However, surface volcanism occurs in a much narrower zone where the melt solidus is intersected at lower crustal depths.

**Supplementary Information:**

The online version contains supplementary material available at 10.1038/s41598-026-44483-8.

## Introduction

**Fig. 1:**
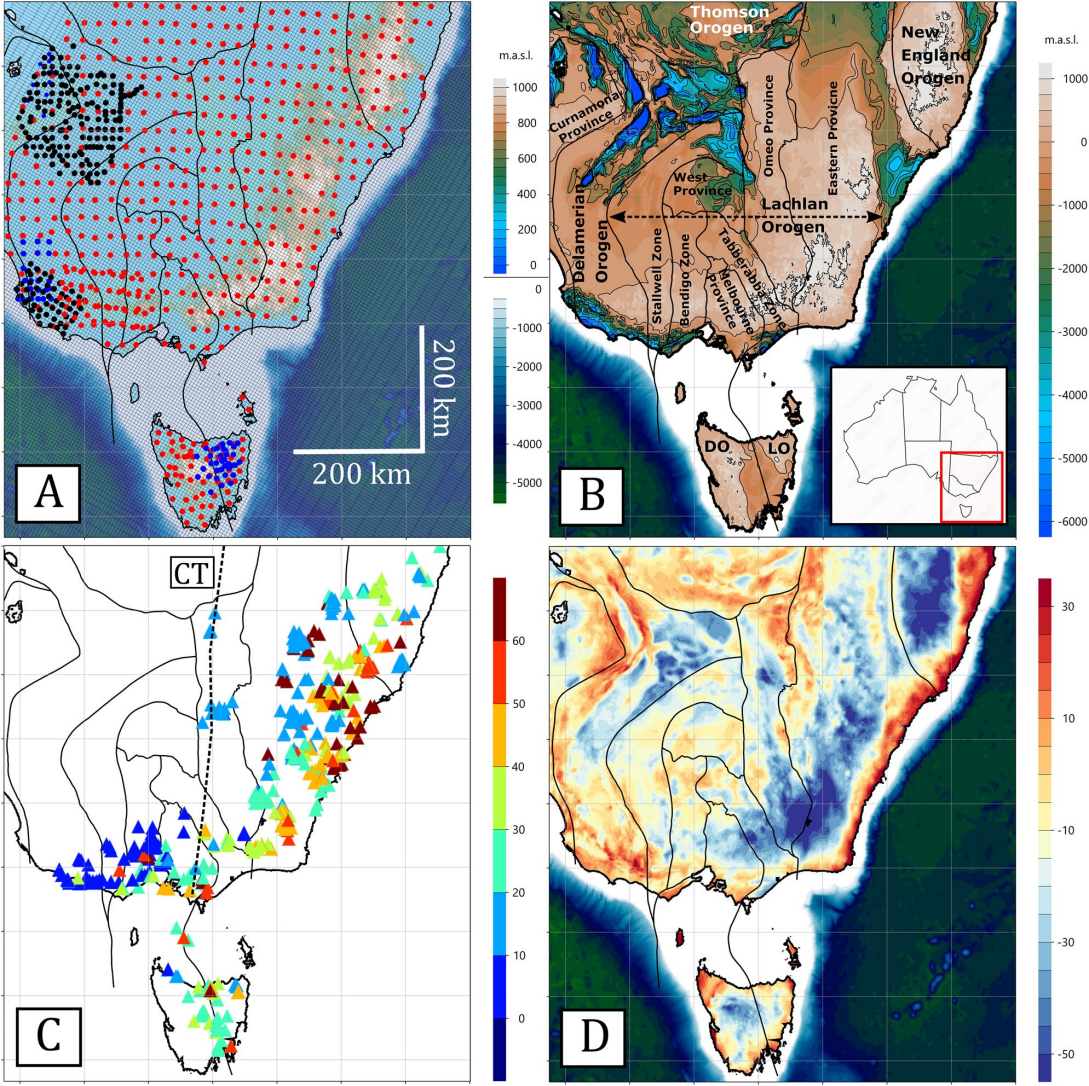
(**a**) Elevation and ocean bathymetry with MT station locations: long-period MT (red), broadband MT (black), and geomagnetic depth sounding (GDS; blue) sites, with primary crustal domains^7^(black lines). The mesh used for 3D inversion, with 10 km inner grid with a strike of 60° is shown in fine lines; (**b**) Elevation of Proterozoic basement and labelled crustal domains; (**c**) Distribution of volcanoes coloured by age in Ma^8^. The dashed line shows the approximate trend of the Cosgrove Hot Spot Track (CT), with volcanoes being progressively younger in a southward direction; (**d**) Bouguer gravity (mGals). Elevation and gravity datasets were obtained from Geoscience Australia’s Geophysical Archive Data Delivery System (GADDS) (https://portal.ga.gov.au/persona/gadds) and are released under the Creative Commons Attribution 4.0 (CC BY 4.0) licence (https://www.ga.gov.au/copyright). Elevation of Proterozoic basement were sourced from Geognostics OZ SEEBASE 2021 (https://www.geognostics.com/oz-seebase-2021), also distributed under a CC BY 4.0 licence. All figures were produced using Viridien Geotools v4.0.3.12574 (https://www.viridiengroup.com/expertise/multiphysics-imaging/geotools) and Inkscape v1.2.1 (https://inkscape.org).

Eastern Australia in Fig. 1a comprises a series of Cambrian to Triassic (550–220 Ma) orogenic systems collectively known as the Tasmanides^1,2^. Competing tectonic models attribute their evolution either to successive terrane accretion events with associated subduction polarity reversals^3^ or to the long-term reactivation and recycling of a continental margin governed by alternating trench advance and retreat^4,5^. The composite orogenic belt consists of five major orogens, namely the Delamerian, Thomson, Lachlan, Mossman (not shown, in Queensland to the north of the area in Fig. 1a), and New England Orogens in Fig. 1b that record the tectonic evolution of the eastern Gondwana margin from the breakup of Rodinia in the Neoproterozoic, accretion of Gondwana and subsequent breakup in the Triassic. As part of the broader Terra Australis Orogen^6^, the Tasmanides formed along the Palaeozoic margins of Gondwana, linking geological provinces that extend from South America through Africa and Antarctica to the Southwest Pacific.

Numerous volcanos are evident along the eastern margin of the Tasmanides^8^ (Fig. 1c). These volcanoes broadly form into two groups. Firstly, the leucitite-bearing Cosgrove Track^9,^^10^ is evident in volcanoes of age 40 − 0 Ma in a hot-spot chain that stretches ~ 2000 km from the oldest in northern Queensland to the youngest in Bass Straits between Victoria and Tasmania^11^. Such volcanoes are not uniformly distributed along the hot-spot chain but are evident at a relatively few discreet locations (as shown in Fig. 1c) suggesting that mantle hot spot fluxes may be modified by the variable thickness of lithosphere that passes over the top at rates of up to 75 km/Ma^9,10^.

The second and significantly more abundant series of lava-field volcanoes have no obvious age progression and extend > 3,000 km from northern Queensland to southern Tasmania^8^. The earliest volcanoes date back ~ 100 Ma coinciding with rifting of the Lord Howe Rise from continental Australia, but eruption frequency has changed markedly with time^11^. Peak eruptive phase was about 20 Ma with up to 80 eruptions/Ma followed by a slight drop to ~ 40 eruptions/Ma, and then a relatively uniform rise to about 60 eruptions/Ma in the last one million years^11^. The volcanic centres exhibit some spatial clustering with age, but with no obvious pattern. The newest volcanoes, shown in dark blue, are in the south-western region known as the New Volcanic Province, with the most recent volcanism about 5000 years ago.

Three main theorems have been advanced to explain the age-independent volcanism: (1) decompression melting from the transition zone due to volatile content from subducted slab stagnation^11^; (2) edge-driven convection at the margins of steps in lithospheric thickness that drive upwelling from volatile-rich upper mantle reservoirs^12^; and (3) melting of low-viscosity pockets of sub-lithospheric mantle due to asthenospheric shear^13^. In this paper we interpret > 800 sites of MT sites shown in Fig. 1a to define a new 3D resistivity model of the lithosphere and asthenosphere for southeastern Tasmanides. Magnetotelluric data have been collected across the Tasmanides over the last forty years, as shown in Fig. 1a. The primary data set (red circles in Fig. 1a) are 530 AusLAMP and legacy long-period sites across South Australia, New South Wales, Victoria, and Tasmania^14–21^. At the time of writing this paper, there were no contiguous AusLAMP sites through southern and eastern Queensland to the north of the array in Fig. 1a. There are two major broadband MT arrays (black circles in Fig. 1a); 154 sites across the Curnamona Province^17^ and 49 sites across the New Volcanic Province in South Australia and Victoria^22^. Although there are many transect broadband MT surveys in South Australia, Victoria and Northern New South Wales, typically along reflection seismic lines, these were not included in the array because the site spacing was smaller than can be reasonably modelled with a grid covering the entire regions. Legacy geomagnetic depth sounding (GDS) sites, for which only vertical magnetic field transfer functions are available, provide additional constraints, including 11 sites in the Curnamona Province^17,23,24^, 24 sites in South Australia^15^, and 35 sites in Tasmania^25,26^.

## Results

**Fig. 2:**
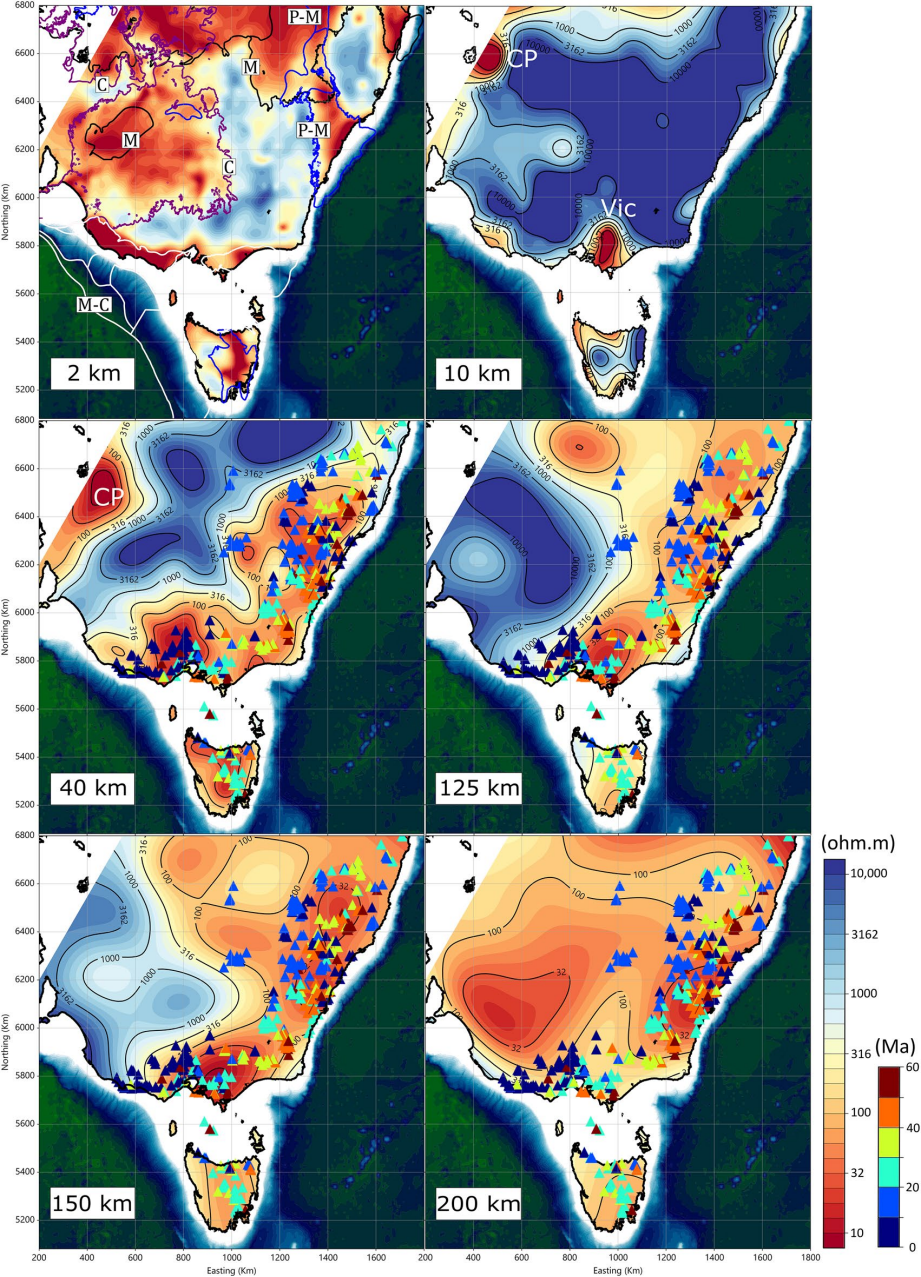
Horizontal slices of the 3-D resistivity model at 2, 10, 40, 125, 150, and 200 km depths. Triangles mark volcano locations^8^, coloured by age (Ma). In the 2 km slice, sedimentary basins are shown as follows; Palaeozoic-Mesozoic (blue lines: P-M); Mesozoic (black lines: M); Mesozoic-Cenozoic (white lines: M-C); Cenozoic (purple lines: C). All panels share the same map extent and coordinate system (UTM Zone 54 S). Sedimentary basin data were sourced from the Geoscience Australia Portal (https://portal.ga.gov.au/) and are copyright Commonwealth of Australia (Geoscience Australia) 2018, released under the Creative Commons Attribution 4.0 International License (CC BY 4.0). All figures were produced using Viridien Geotools v4.0.3.12574 (https://www.viridiengroup.com/expertise/multiphysics-imaging/geotools) and Inkscape v1.2.1 (https://inkscape.org).

A new three-dimensional resistivity model of the Tasmanides is shown in Fig. 2, derived from an inversion of all MT sites and GDS data. The inversion uses period bands of 10–10,000 s for long period MT, 10 − 1,000 s for broadband MT, and 1,000–10,000 s for GDS. The model grid compromises 157, 223 and 114 cells in the x, y, and z directions, respectively, and is rotated 30 degrees clockwise. The core model region employs a uniform cell size of 10 km, as shown in Fig. 1a. Three-component magnetic (x, y and z) and two-component electric field (x and y) data were used for long-period MT, whereas only horizontal magnetic and electric field components (x and y) were used for broadband MT; GDS data include only the three-component magnetic fields. The final RMS was 1.92. Details of the 3D inversion are provided in the Methods section, and additional images of data fits from the 3D inversion are provided in a Supplementary Section. The model in Fig. 2 is similar to previously published models^18,^^19^, but includes more MT and GDS sites over a wider area. Additionally, the 3D inversion approach implemented in Viridien Geotools in our paper tends to produce smoother models at depth^27^.

At 2 km depth, the resistivity structure is displayed with a dynamic range of 10 to 10,000 Ohm.m, together with outlines of Palaeozoic to Cenozoic sedimentary basins. Regions of markedly low resistivity (~ 10 Ohm.m) show a clear spatial correspondence with major basins, consistent with the enhanced porosity and fluid content characteristic of shallow sedimentary sequences.

To illustrate the principal lithospheric transitions, resistivity slices are also shown at five additional depths: upper crustal (10 km), lower crustal (40 km), and three representative mantle depths (125, 150 and 200 km). The upper crustal section at 10 km depth is predominantly resistive, with most regions exceeding 1,000 Ohm.m, as expected for relatively cool (< 300 °C), felsic crustal compositions^28^. Two exceptions stand out: (a) a conductive zone along the eastern margin of the Curnamona Province (CP), and (b) a second conductive feature located between Victoria and Tasmania (Vic). Previous studies^17,24^ have attributed the upper-crustal conductor in the Curnamona Province to the presence of graphite within Palaeoproterozoic metasediments. However, the origin of the conductor in Victoria has not been previously noted^15^.

At 40 km depth, approximately corresponding to the base of the crust in eastern Australia^29^, both age-dependent and age-independent volcanic centres coincide with regions of reduced resistivity (< 100 Ohm.m). The Curnamona Province maintains its conductive character (CP) at this level, suggesting a lithologically or thermally controlled deep crustal source of reduced resistivity.

At mantle depths of 125 km and 150 km, a strong spatial correlation emerges between the volcanic fields and low-resistivity domains (< 100 Ohm.m). These depths also exhibit pronounced lateral resistivity gradients near the 1,000 Ohm.m contour, indicative of sharp transitions in mantle composition, temperature, or volatile content. Volcanoes associated with the Cosgrove Track show weaker alignment with these conductive regions, implying a more complex or distributed mantle source beneath that system. By 200 km depth, the resistivity structure becomes comparatively homogeneous at ~ 100 Ohm.m. Although this value approaches a typical half-space resistivity, MT sensitivity at most sites extends well beyond 200 km^18^, and thus the observed uniformity likely represents a genuine large-scale property of the sub-lithospheric mantle rather than an artefact of depth-limited resolution.

**Fig. 3:**
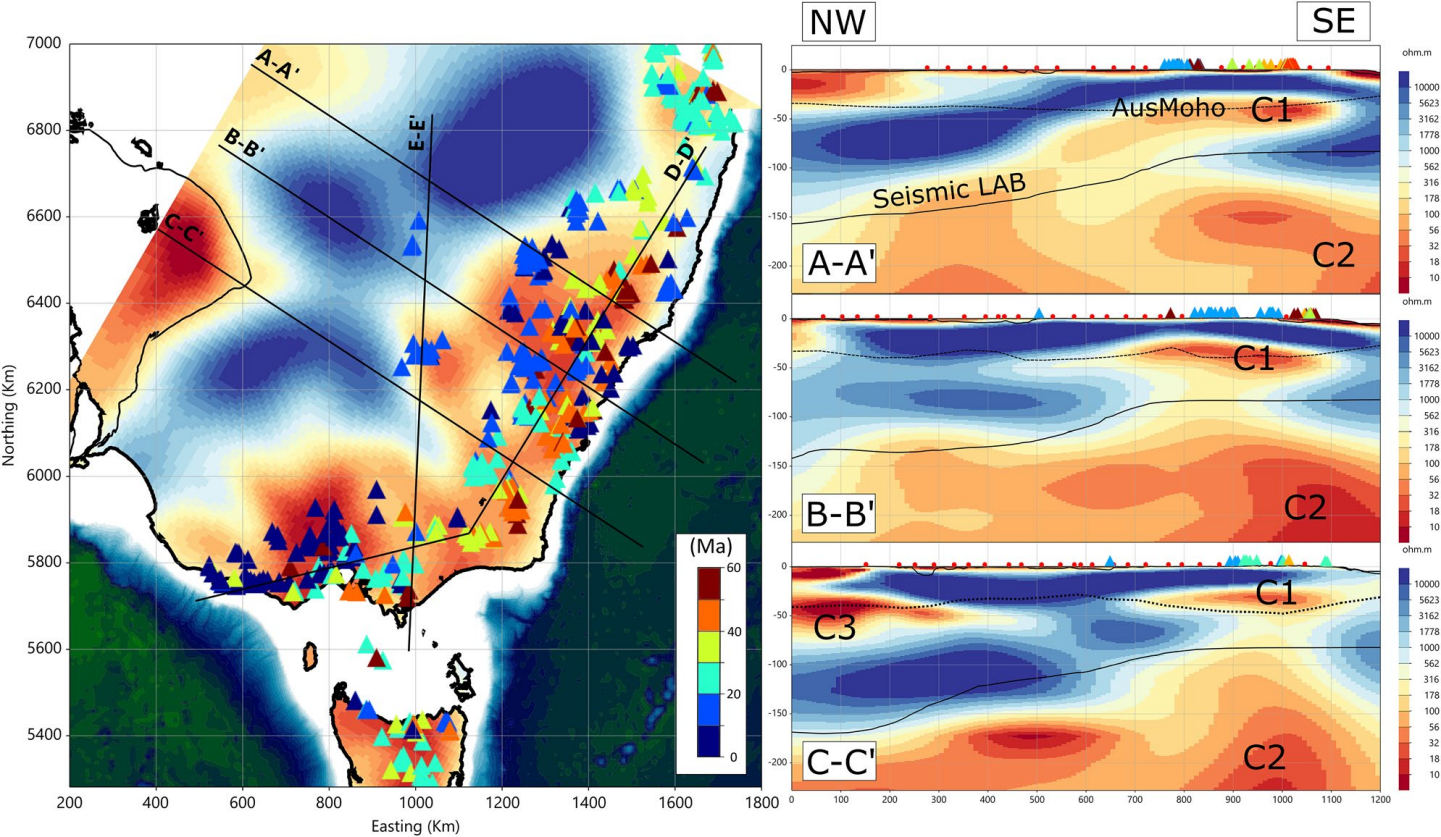
Integrated plan–section view of the resistivity model. Left: Resistivity map at 40 km with volcano locations and five profiles (A–A′, B–B′, C–C′, D–D′, E–E’). Right: West–east resistivity cross-sections along A–A′, B–B′, and C–C′. Red circles denote neighbouring magnetotelluric stations, providing a visual guide to the model’s lateral resolving power. Coloured triangles mark volcano locations. The dashed line indicates the Moho estimate^29^, and the solid line shows the estimated seismic lithosphere–asthenosphere boundary^30–32^. All panels share the same resistivity colour bar (Ohm.m). All figures were produced using Viridien Geotools v4.0.3.12574 (https://www.viridiengroup.com/expertise/multiphysics-imaging/geotools) and Inkscape v1.2.1 (https://inkscape.org).

**Fig. 4:**
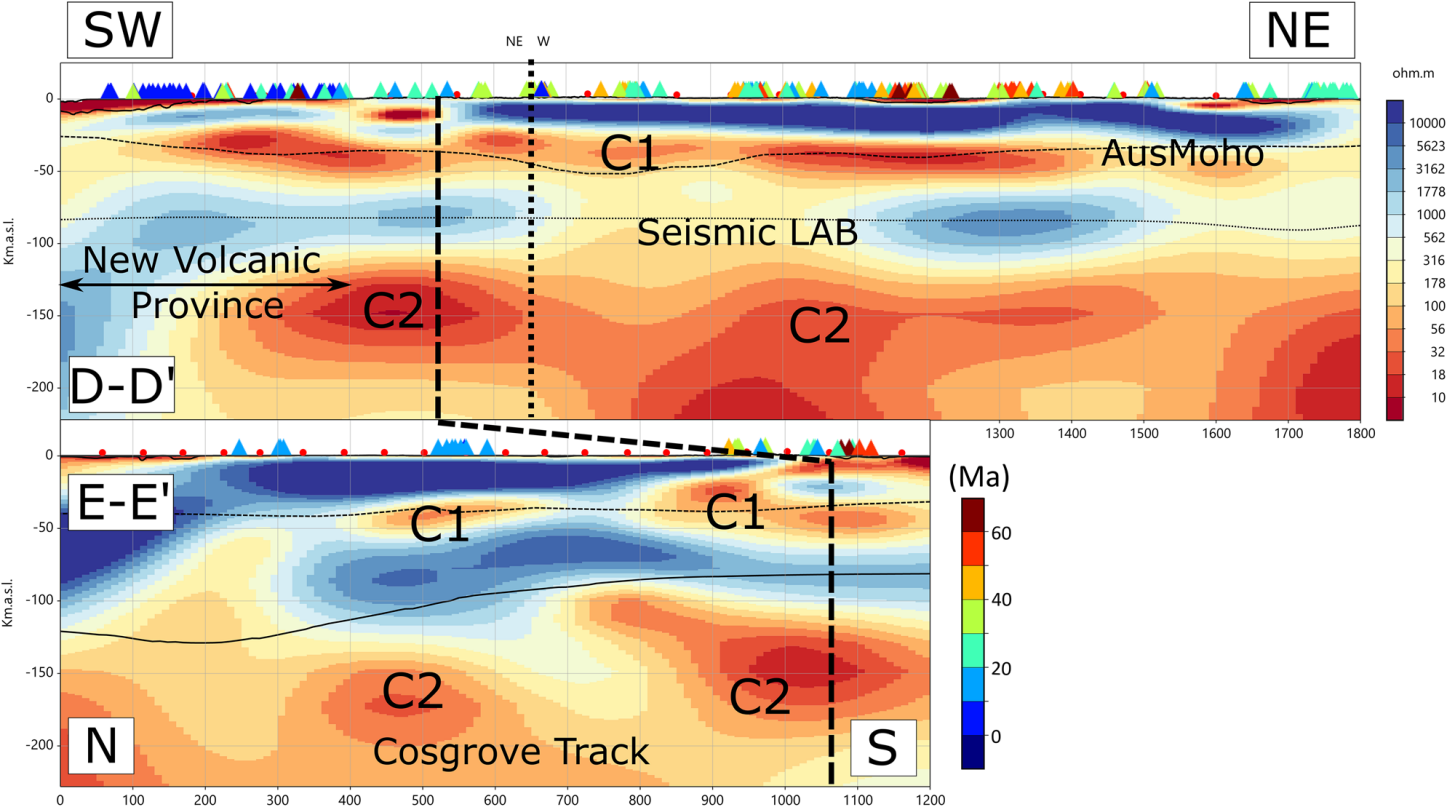
Resistivity cross-sections along D–D′ (Cenozoic volcanos, including the New Volcanic Province) and E–E′ (Cosgrove Track). Red circles denote neighbouring magnetotelluric stations. Coloured triangles mark volcano locations coloured by age shown by the scale bar 0–60 Ma. The fine-dashed horizontal line indicates the Moho^29^; the solid line shows the LAB^30–32^ as for Fig. 3. The thick-dash vertical line shows where profiles D-D’ and E-E’ intersect; the thin-dash vertical line in D-D’ shows the change in orientation of the profile. All figures were produced using Viridien Geotools v4.0.3.12574 (https://www.viridiengroup.com/expertise/multiphysics-imaging/geotools) and Inkscape v1.2.1 (https://inkscape.org).

In Figs. 3 and 4, we present a series of lithospheric resistivity cross-sections extending to a depth of 250 km. The left-hand panel in Fig. 3 shows the locations of these sections superimposed on a resistivity map at 40 km depth. The finer black outline at the western end of cross-section C–C′ indicates the extent of the Curnamona Province and Adelaide Rift Complex, as shown in Fig. 1b. Cross-sections A–A′, B–B′ and C–C′ each span 1,200 km from the continental interior toward the coast, oriented roughly perpendicular to the strike of the Cenozoic volcanic chain and to the elevation of the Great Dividing Range (Fig. 1a), but oblique to major orogenic boundaries. These sections are spaced ~ 200 km and trend NW–SE. Magnetotelluric coverage is incomplete along the northeastern end of A–A′. For all sections, we also include the estimated Moho depth^29^ and the depth to the seismically defined lithosphere–asthenosphere boundary (LAB)^30–32^. Volcanoes within a 40-km corridor of each cross-section trace are projected onto the lines.

A consistent feature across all three inland-to-coastal transects is that age-progressive volcanic centres overlie a region of low-resistivity (< 100 Ohm.m) lower crust, denoted C1. This conductor is typically ~ 300 km wide and appears confined to the lower crust rather than extending into the underlying mantle. Within the Curnamona Province at the western end of C–C′, conductor C3 comprises both an upper-crustal low-resistivity region and a very low-resistivity (< 10 Ohm.m) zone at or below the Moho. A similar multi-level conductive structure in this region was previously identified^17^, attributed to graphite-rich Paleoproterozoic suture material, and a deeper, and extensive conductor that reflects mantle-derived carbon emplaced during Neoproterozoic extension associated with Rodinia breakup (~ 800 Ma).

The mantle lithosphere between the Moho and approximately 125 km depth is generally resistive (> 1,000 Ohm.m). Immediately beneath conductor C1, resistivity gradients are smoothed by inversion regularisation, making precise values difficult to resolve. Nevertheless, the deeper mantle (> 150 km) is characteristically conductive, forming a region (C2) with resistivities near ~ 10 Ohm.m. In all three cross-sections, upper-mantle resistivity increases toward the continental interior, suggesting a spatial and possibly genetic link between crustal conductor C1 and mantle conductor C2. Below ~ 200 km depth, the upper mantle becomes predominantly conductive (< 50 Ohm.m).

Two additional sections in Fig. 4, at the same vertical and horizontal scale as Fig. 3, emphasise the relationship between lithospheric resistivity and the distribution of Cenozoic volcanism. Cross-section D–D′ is ~ 1,800 km in length and traverses the full extent of the volcanic chain. The southwestern 400 km corresponds to the New Volcanic Province, the youngest volcanic field, active within the past 10 Ma^22^. A change in strike occurs at ~ 700 km along the profile, indicated by a finer dashed line. The resistivity structure along D–D′ is comparatively uniform: the upper 20 km is highly resistive (> 10,000 Ohm.m), the lower-crustal conductor C1 lies at 30–50 km depth consistent with independent Moho estimates, and conductor C2 appears at ~ 150 km depth across most of the transect. However, beneath the New Volcanic Province, C2 becomes notably more resistive, consistent with suggestions that enhanced edge-driven convection may have influenced melting and volcanism in this region^22^.

Profile E–E′, extending ~ 1,200 km, follows the trace of the Cosgrove Track and intersects D–D′ at the location marked by the larger dashed line. Volcanoes along this profile exhibit partial spatial correlation with lower-crustal conductor C1, although the crust generally appears more resistive than along D–D′. Notably, volcanic centres at distances of 500–600 km coincide with both an enhanced lower-crustal conductor and the upper portion of mantle conductor C2, suggesting a coupled crust–mantle control on melt generation along this segment of the track.

## Discussion

**Fig. 5:**
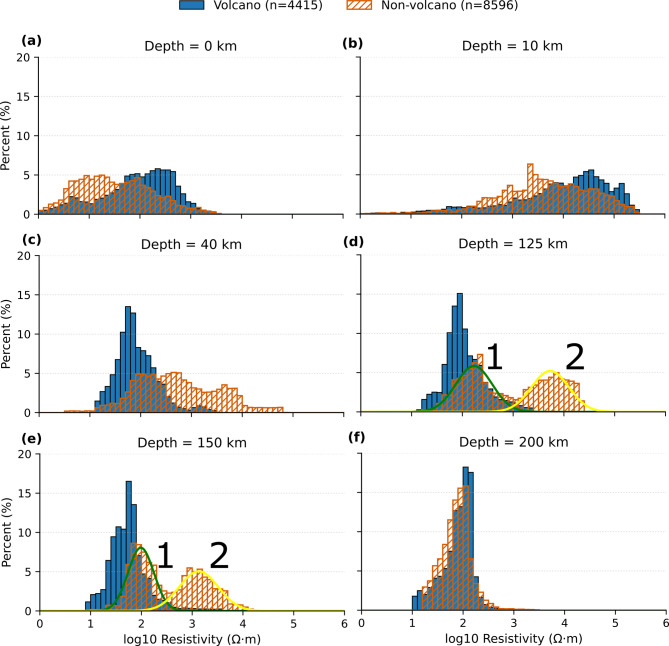
Depth-dependent resistivity distributions for volcano (blue) and non-volcano (orange) regions at 0, 10, 40, 125, 150, and 200 km. Histograms are normalized to percentage. The x-axis is log₁₀ resistivity (Ohm.m); the y-axis is frequency (%). A common binning scheme is used across depths with Δlog₁₀ρ = 0.1. All figures were produced using Python v3.12.7 (https://www.python.org/) and Inkscape v1.2.1 (https://inkscape.org).

We use the location of the volcanos to define resistivity beneath grid-nodes that are either within a 40 km radius of a volcano (a total of 4415 nodes for 478 volcanoes) or (b) more than 40 km from a volcano, but still within the footprint of MT sites in Fig. 1a (a total of 8596 nodes). The resistivity values at depths of 0, 10, 40, 125, 150 and 200 km are then presented as histograms, as shown in Fig. 5 and summarised in Table 1. A radius of 40 km from a volcano was arbitrary but was chosen on the premise that the melt region would most likely be significantly wider than the vent.

At the surface, non-volcano nodes are generally lower in resistivity than for volcano nodes by about an order of magnitude, but with a very wide range. This is primarily because the volcanoes are typically on elevated terrain of the Great Dividing Range with little sedimentary cover (as shown in Fig. 1a and c), whereas the non-volcano nodes are often on sedimentary basins, as shown in Fig. 1b. At 10 km, all nodes are more resistive by about two orders of magnitude, and volcano nodes are again more resistive.

**Table 1:** Volcano and non-volcano distributions by depth: log₁₀ ρ (Ohm.m) mean and standard deviation.

Depth (km)	Volcano (Mean)	Volcano (STD)	Non-Volcano Mean (Peak 1)	Non-Volcano STD (Peak 1)	Non-Volcano Mean (Peak 2)	Non-Volcano STD (Peak 2)
40	1.92	0.41	2.72	0.80	-	-
125	2.04	0.40	2.23	0.36	3.73	0.37
150	1.75	0.38	1.99	0.25	3.13	0.38
200	1.89	0.31	1.86	0.30	-	-

At 40 km depth in the lower crust, volcano nodes are significantly less resistive, with a mean of 1.92 log units (83 Ohm.m) with a standard deviation of 0.41 log units, compared to the non-volcano nodes that have a mean of 2.72 (524 Ohm.m) and standard deviation of 0.80. The non-volcano nodes suggest a slightly bimodal distribution.

In the upper mantle, volcano sites similarly show a narrow distribution, with means of 2.04 (109 Ohm.m) at 125 km, and 1.75 (56 Ohm.m) at 150 km depth respectively, with similar standard deviations of ~ 0.4 log units. By contrast, the non-volcano nodes show a bimodal distribution, suggesting that these nodes are above significantly different lithosphere-asthenosphere structures. At 125 km, using a two-Gaussian distribution model, the means are 2.23 (169 Ohm.m) for the Gaussian curve denoted 1 and 3.73 (5,370 Ohm.m) for the Gaussian curve denoted 2, while at 150 km, the means are 1.99 (98 Ohm.m) and 3.13 (1,348 Ohm.m). By 200 km, the distributions are identical with a mean of 1.86 (72 Ohm.m) and a small standard deviation suggesting that the sub-lithosphere has uniform resistivity.

Spatial variations in mantle resistivity at 125 and 150 km depth reveal two statistically distinct distributions, indicating that the upper mantle beneath eastern Australia is not characterised by a single continuum of physical properties. Instead, the resistivity structure is bimodal, reflecting variations in composition, temperature, and hydration state. These observations suggest that the lithospheric mantle contains discrete domains with contrasting thermo-chemical characteristics rather than a smoothly varying gradient.

**Fig. 6:**
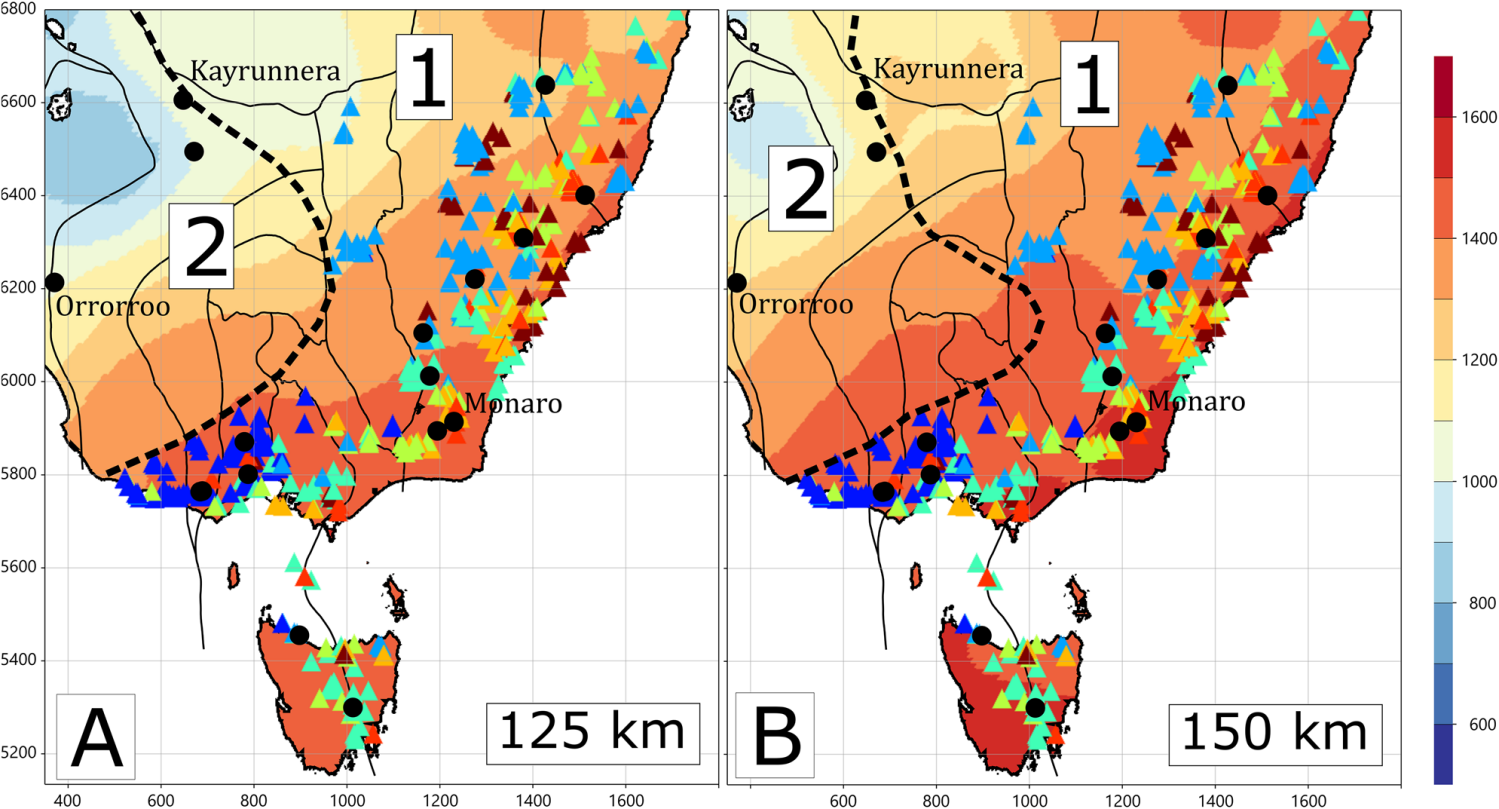
Distribution of resistivity at depths of 125 and 150 km from two Gaussian distributions denoted 1 and 2 in Fig. 5. The dashed line shows the approximate division between the two distributions. Also shown are volcanoes coloured by age (triangles), location of mantle xenoliths (black circles), on a predicted temperature map in °C^33^. All figures were produced using Viridien Geotools v4.0.3.12574 (https://www.viridiengroup.com/expertise/multiphysics-imaging/geotools) and Inkscape v1.2.1 (https://inkscape.org).

To investigate the spatial expression of these domains, Fig. 6 maps the geographic distribution of the two resistivity populations alongside predicted mantle temperatures derived from palaeo-geotherms constrained by mantle xenoliths and the FR12 seismic model^32,33^. Numbered regions correspond to the two Gaussian distributions identified at 125 and 150 km depth in Fig. 5 (noted as 1 and 2) and are separated by the dashed boundary. Also shown are the locations of mantle xenolith^33^ and Cenozoic volcanic centres coloured by age^8^. The map demonstrates that the transition between the two resistivity regimes broadly coincides with the predicted thermal structure of the mantle, although the temperature field is inherently smoothed owing to the sparse and spatially uneven xenolith constraints.

Taken together, these results imply that mantle resistivity varies in a step-like manner from west to east, rather than forming a unimodal or gradational distribution. This sharp transition likely marks a lithospheric boundary separating two domains with distinct physical properties. A similar step in lithospheric thickness is evident in the FR12 seismic model^30^,^32^, estimates of the lithosphere-asthenosphere boundaries in Figs. 3 and 4. The location of the lithospheric step is several hundred kilometres east of the Tasman Line^34^ which broadly corresponds to the margin of Proterozoic Australia, suggesting that it is not simply a compositional change. The youngest volcanism in the New Volcanic Province (0–10 Ma; dark blue symbols in Fig. 6) occurs adjacent to this boundary, consistent with a role for edge-driven convection in focusing magmatism^22^. The easternmost projection of this lithospheric step also aligns with the leucitite-bearing volcanism of the Cosgrove Track, suggesting that the boundary may exert a first-order control on both the location and style of intraplate volcanic activity^9,10^.

**Fig. 7:**
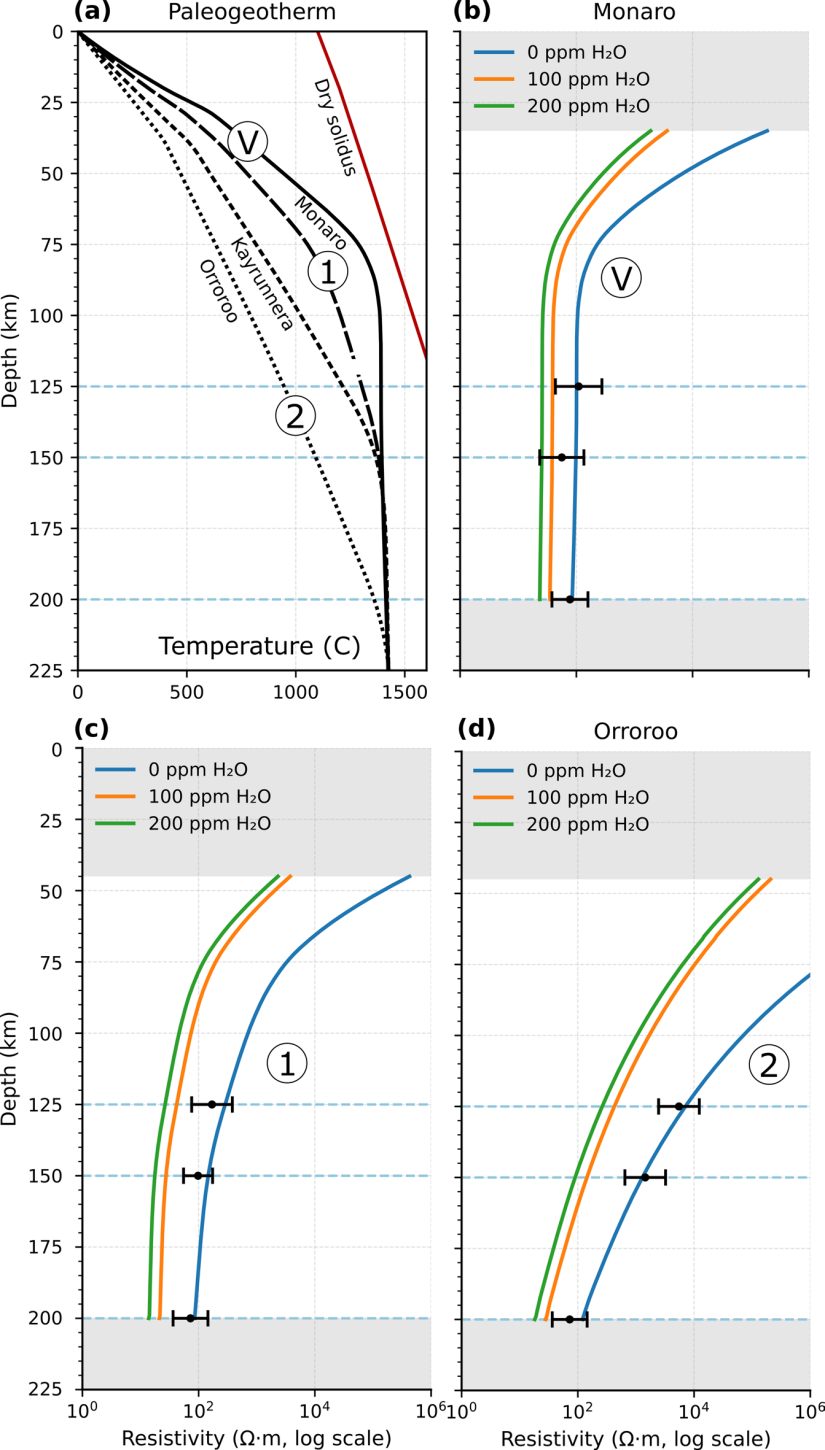
Thermal–electrical structure for three representative regions: Monaro (volcanic), “Unnamed” (non-volcanic region 1), and Orroroo (non-volcanic peak-2). (**a**) Palaeo-geotherms for Monaro, Kayrunnera, Orroroo, and a constructed “Unnamed” geotherm defined as the arithmetic mean of Monaro and Kayrunnera, plotted against the dry solidus^33^. (**b–d**) Predicted mantle resistivity profiles derived with MATE^35^ from the panel-(**a**) geotherms using a lherzolite parameterization for three bulk H₂O contents (0, 100, 200 ppm; blue, orange, green). Black symbols with horizontal error bars show MT mean resistivity ± 1 SD for volcano and non-volcano domains (from Figs. 5 and 6). Grey shading marks depth ranges with limited constraint (35–200 km for volcanic region and 45–200 km for non-volcanic region). Axes: depth (km) vs. resistivity (Ohm.m, log scale). All figures were produced using Python v3.12.7 (https://www.python.org/) and Inkscape v1.2.1 (https://inkscape.org).

To understand the cause of mantle resistivity across the Tasmanides, we examine the temperature-resistivity dependence using the Mantle Analysis Tool for Electromagnetics (MATE) software^35^. In Fig. 6 we show that the distribution of mantle xenoliths is quite sparse, with many xenoliths occurring in the volcano regions, but few to the west. To generate three representative temperature distributions in Fig. 7a, we have chosen the Monaro xenolith as representative of the volcano nodes (V); Orroroo in the far west as representative of temperature in region 2 of Fig. 6; arithmetically average temperatures of Monaro and Kayrunnera (on the boundary between regions 1 and 2) as representative of region 1 in Fig. 6. The temperature curves are palaeo-geotherms but are assumed to be broadly consistent with the current thermal state of the mantle given the active volcanism over the last 100 Ma. All four curves in Fig. 7a converge to the mantle adiabat ~ 1,400 °C at depths ranging from 75 km for Monaro, to > 200 km for Orroroo, indicating profound differences in thermal lithosphere thickness from east to west.

We compute predictive resistivities for these mantle geotherms based on a simple lherzolite composition (63% olivine, 30% orthopyroxene, 7% clinopyroxene) using MATE software^35^ for dry lherzolite (0 ppm H₂O) and realistic hydration of olivine (100 and 200 ppm H₂O; Fig. 7b–d). Modelled mantle resistivities at 125–200 km (black points with one standard deviation uncertainty) for the volcano nodes in 7b, region 1 nodes in Fig. 7c and region 2 nodes in Fig. 7d align with the dry-olivine curves across all three domains. Hydrated mantle resistivity predictions are systematically higher than observed by ~ 0.5–1 log unit.

A lherzolite composition was chosen as a conservative option given few xenolith constraints for most of the Tasmanides. Partial melting of the mantle would typically lead to depletion to harzburgite (73% olivine, 25% orthopyroxene, 2% clinopyroxene) and alternatively metasomatism may enhance clinopyroxene content to wehrlite (71% olivine, 2% orthopyroxene, 27% clinopyroxene). We show in the Supplementary that the MATE predicted mantle resistivities vary by less than 0.2 log units of resistivity for lherzolite-harzburgite- wehrlite so that that the results in Fig. 7 are not specifically dependent on mantle composition.

The lateral ranking of temperatures explains the residual differences: the hottest geotherm beneath Monaro yields the lowest resistivities, the broad region is slightly cooler and more resistive, and the western Orroroo domain is coldest and most resistive. Together, these relationships imply limited mantle hydration (≲ O(10¹) ppm) across the whole of the Tasmanides, a primarily thermal control on upper-mantle resistivity, implying a lithospheric step expressed as warmer, thinner lithosphere beneath region 1 and the volcanoes in Fig. 6 relative to the cooler western component.

The lower continental crust is widely interpreted to consist predominantly of mafic granulitic lithologies, particularly beneath tectonically stable regions^28^. These rocks are characterized by assemblages dominated by clinopyroxene, orthopyroxene and plagioclase, with mineralogies that are nominally anhydrous^36^. Independent constraints from xenolith thermos-barometry, surface heat-flow observations and seismic imaging indicate that temperatures in this domain typically range between ~ 700 and 1,000°C^33^. Compared with upper-mantle peridotites, lower-crustal pyroxenes are systematically enriched in Fe, while plagioclase in the lower crust exhibits higher Na contents than plagioclase in shallow, felsic crustal rocks^28^. Although nominally dry^37^, lower-crustal minerals generally incorporate water as hydrogen-related point defects, with estimated concentrations from < 100 to > 1,000 ppm H₂O by weight, commonly exceeding those inferred for the upper mantle. Xenoliths of granulitic composition entrained in volcanic eruptions further indicate that the lower continental crust is internally stratified, comprising alternating layers enriched in pyroxene and plagioclase^38^.

**Fig. 8:**
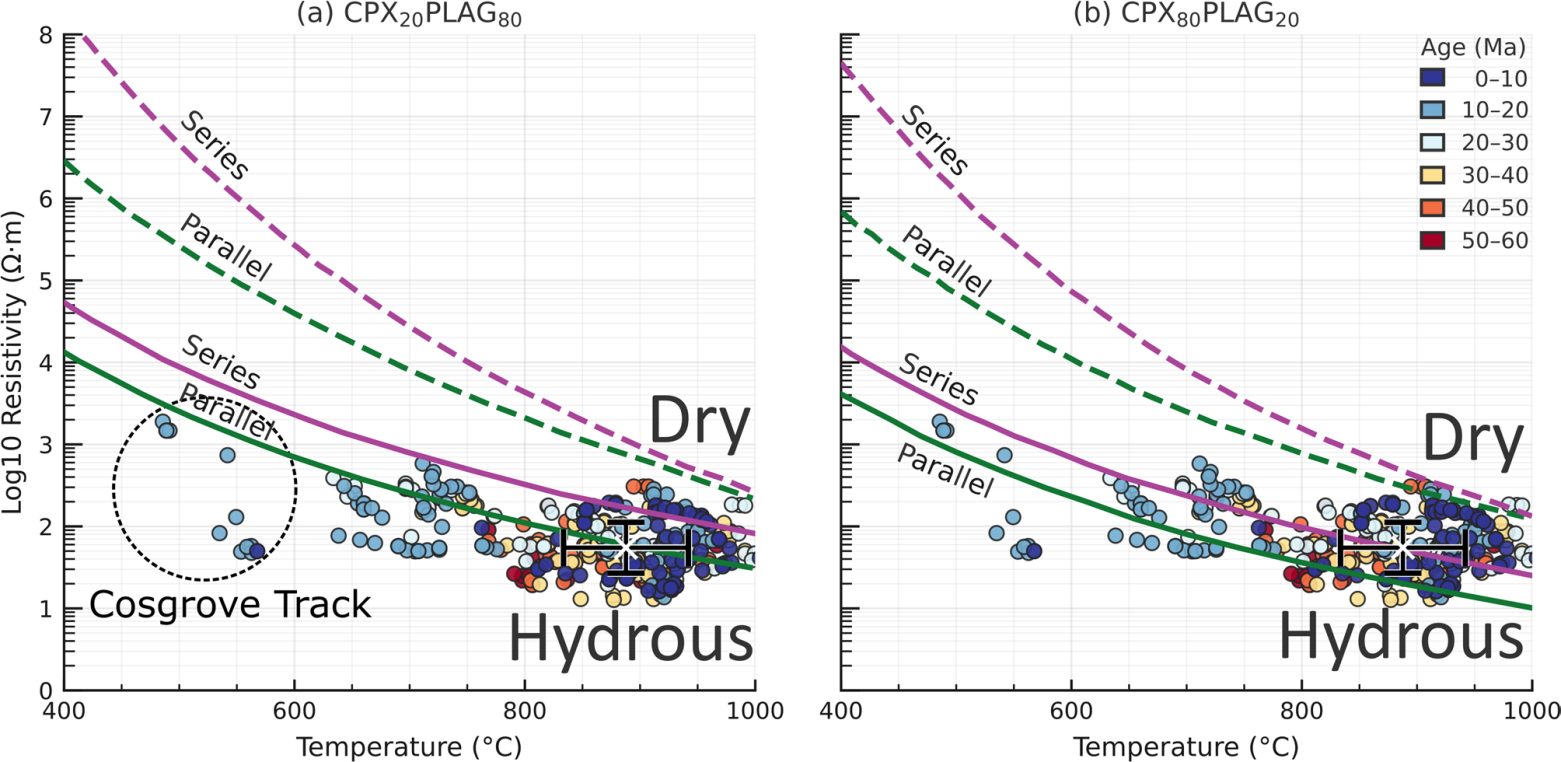
Estimated bulk electrical resistivity of lower-crustal rocks for (left) plagioclase-rich and (right) pyroxene-rich compositions, modified from refs^28,39,40^. Solid curves represent hydrous conditions; dashed curves represent dry conditions. Circles show volcano sites coloured by eruptive age (Ma) and plotted at their MT-inferred resistivity and temperature in 40 km depth. Axes: temperature (°C) versus resistivity (Ohm.m; log scale). All figures were produced using Python v3.12.7 (https://www.python.org/) and Inkscape v1.2.1 (https://inkscape.org).

The expected relationships between electrical resistivity and temperature for representative end-member lithologies, specifically (a) a plagioclase-dominated layer and (b) a clinopyroxene-dominated layer are shown in Fig. 8. These curves are derived from published petrophysical constraints^28,38^, for both anhydrous and hydrous conditions using series and parallel mixing bounds. For the hydrous scenarios, water contents of 400 ppm H₂O in clinopyroxene and 250 ppm H₂O in plagioclase are assumed, consistent with experimentally and observationally constrained ranges for lower-crustal minerals.

From the 3D resistivity model, we extracted resistivity values at 40 km depth beneath the nodes closest to volcanic centres, yielding 408 estimates. Corresponding temperatures at the same depth were obtained from the regional thermal model^33^, which integrates seismic constraints and xenolith data. Both the resistivity model and the temperature estimates are necessarily smoothed and regularized representations of the subsurface, reflecting limitations imposed by data coverage and model parameterization. Nevertheless, the large number of samples provide a statistically robust characterisation of resistivity–temperature conditions in the lower crust beneath the volcanic provinces.

The resulting resistivity–temperature pairs plotted in Fig. 8 define a mean temperature of 887 ± 54 °C and a mean log₁₀ resistivity of 1.74 ± 0.31 (55 Ohm.m), excluding the Cosgrove Track sites, for which the model suggest temperatures of < 600°C^33^. The cross-section in Fig. 4 shows that a low-resistive lower-crustal region is also present beneath the Cosgrove Track volcanoes, suggesting that there may be more localised high temperatures from the locus of a hot spot and/or edge-driven convection at the lithospheric step.

When compared with compositional end-member models of clinopyroxene–plagioclase compositions and their series/parallel bounds, the observations consistently lie within the hydrous field and fall below predictions for dry lower crust, regardless of the assumed mineral proportions. This systematic offset requires the presence of modest hydration, most probably through hydrous mineral phases such as amphibole or mica^38^. Although the present data do not resolve the detailed microstructural arrangement of conductive pathways, the coherence of the observations across a range of compositions indicates that hydration exerts a first-order control on lower-crustal resistivity beneath the volcanic regions, rather than bulk mineralogy alone.

**Fig. 9:**
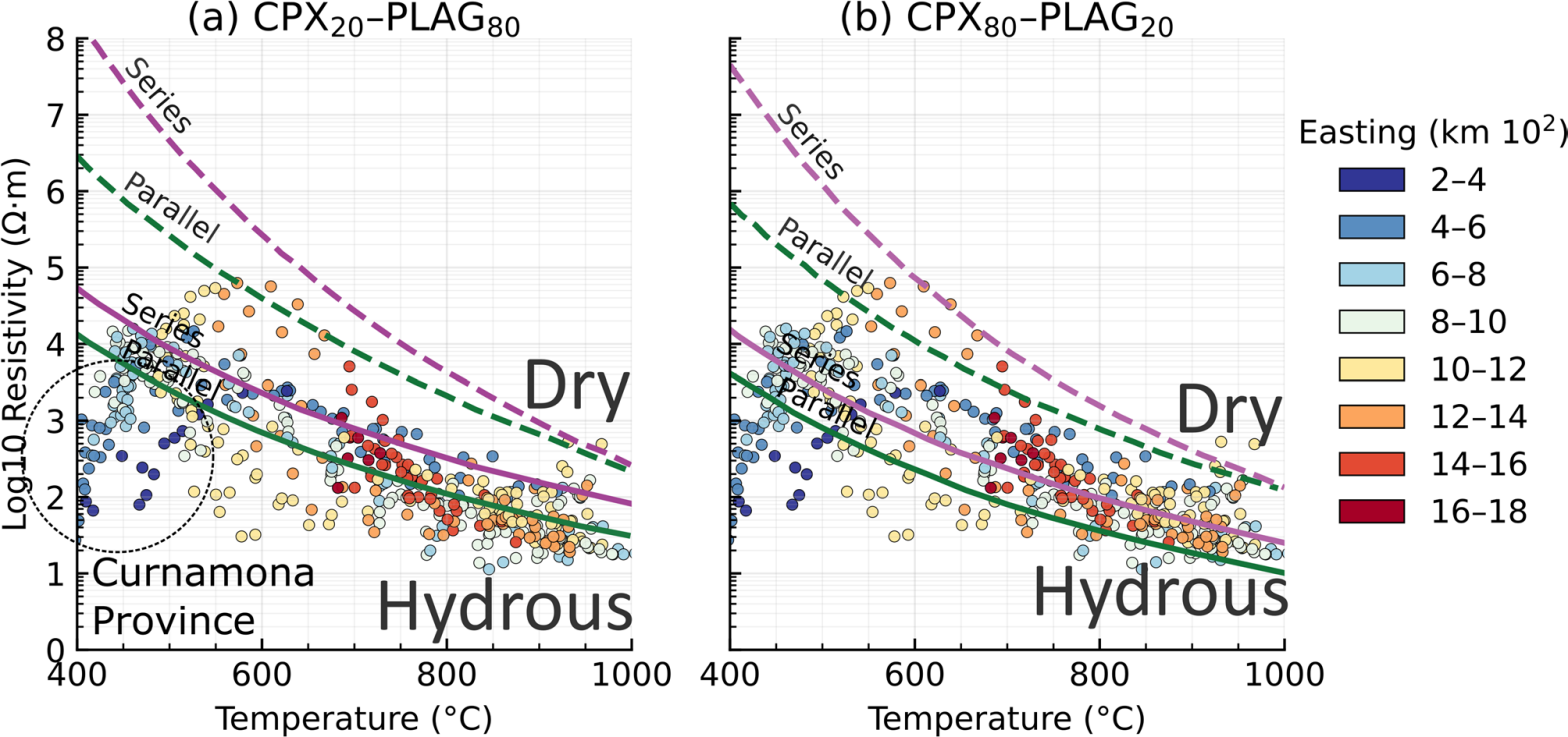
As in Fig. 8, with magnetotelluric station samples highlighted. Circles mark MT site locations and are coloured by easting (UTM, km). Each site is annotated by its resistivity at 35 km (log₁₀ ρ, Ohm.m) and temperature at 40 km (°C), as indicated in the legend. All figures were produced using Python v3.12.7 (https://www.python.org/) and Inkscape v1.2.1 (https://inkscape.org).

We extend the analysis to all continental nodes from the 3D model in Fig. 9. Nodes are colour coded by eastings (in UTM Zone 54 S projection). The resistivity-temperature estimates for each node show a similar trend to the hydrous curves over the temperature range of 400–1,000 °C, with almost all points being below the dry mineralogy curves.

The nodes in the Curnamona Province in the western side of the grid (darker blue colours) are anomalously conductive, with resistivity ~ 100 Ohm.m compared to the predicted hydrous resistivity curves at 400–500 °C of ~ 10,000 Ohm.m. There is no evidence for high temperature in the lower crust as the Orroroo xenolith palaeo-geotherm in Fig. 7 indicates temperatures < 500 °C at 50 km depth, and it is unlikely that additional hydration of crustal minerals would be sufficient. Thus, an additional conductive mechanism is required, potentially graphite from mantle sources of carbon flux during the extension and rifting of the Curnamona Province^17,18,24^.

## Conclusion

A regional 3D resistivity model of the Tasmanides orogenic systems in eastern Australia indicates a step in lithospheric properties between volcanic and non-volcanic domains, expressed by distinctly bimodal resistivities at 125–150 km that converge by ~ 200 km. The contrast is best explained by temperature: volcanic provinces sample mantle that approaches or locally exceeds the solidus, whereas large adjacent regions remain hot yet sub-solidus, consistent with broadly elevated geotherms and decompression melting distributed above a stagnated slab segment. Resistivity–temperature modelling favours a dry upper mantle, implying that fluids are largely extracted during melting and may instead contribute to mafic underplating at the base of the crust. The absence of volatiles in the upper mantle suggests that asthenospheric shear due to changes in viscosity is not the primary cause of volcanism. However, the existence of a step in lithospheric resistivity and thus thermal structure and its spatial correlation with the New Volcanic Zone and the leucite Cosgrove Track volcanoes suggest that edge-driven convention may localise and focus melt migration.

In the lower crust at 40 km beneath volcanoes, temperatures of 800–1,000 °C (mean 887 ± 54 °C) with log₁₀ ρ ≈ 1.74 ± 0.31 (~ 52 Ohm.m) require modest hydrous phases, whereas comparable evidence for hydration is weaker in non-volcanic areas. The lower crust beneath the volcanoes is metasomatized/underplated by decompression melt, and at temperatures > 800 C lies close to the melt solidus and the percolation threshold. Thus, small perturbations of metasomatism can lead to crustal melting and surficial volcanism that has no age progression.

## Methods

A composite dataset was assembled by combining AusLAMP MT sites and legacy long period MT sites^14–21^, broadband MT sites^17,22^ and legacy geomagnetic depth sounding GDS measurements^15,17,23–26^ (Fig. 1). The resulting compilation comprises 530 long-period MT responses (10–10,000 s), 203 broadband MT responses (10–1000 s), and 70 GDS responses (1000-10,000 s), distributed over ~ 1,550 × 1,450 km.

Frequency-domain transfer functions for LP-MT, BB-MT and GDS were estimated with the BIRRPS codes^41,42^ and subsequently curated using MTPy^43^ and Viridien Geotools. The LP-MT time series were acquired at 10 Hz over ~ 4–6 weeks per site using two configurations: LEMI-424 systems (AuScope/Geoscience Australia) with fluxgate magnetometers and two 50–100 m electric dipoles terminated by Ag–AgCl electrodes, or EarthData Logger units paired with Bartington Mag-03 fluxgate sensors, with two 50 m dipoles and Ag–AgCl or Pb–PbCl electrodes. The broadband MT data were acquired using AuScope LEMI-423 systems, equipped with LEMI-120 horizontal induction coils and orthogonal 50 m electric dipoles with Ag–AgCl electrodes, as well as AuScope Phoenix MTU-5 C instruments^17^. Data were typically recorded for two to four days per site, with sampling rates of 1000 Hz for the LEMI-423 systems and 20,000 Hz (burst mode) for the MTU-5 C instruments. Geomagnetic Depth Sounding measurements employed magnetometers developed at Flinders University and University of Tasmania, deployed for several months at 1-min sampling with ~ 1 nT resolution^15,17,23–26^. To assess subsurface large-scale resistivity structure while remaining insensitive to galvanic distortion in electric field measurements^44,45^, phase tensor plots of the data are shown in Supplementary Figure S1.

Three-dimensional inversions were performed in Viridian Geotools using finite-difference forward modelling and a nonlinear conjugate-gradient scheme^46,47^. The computational mesh employed a 10 km inner grid encompassing all stations in Fig. 1, with geometric padding (growth factor 1.20 between adjacent padding cells) extending 1,000 km to the south and east. Vertically, an air layer of 25 m (no topography) was followed by cells increasing in thickness by 5% per layer to 40 km (Moho) and thereafter by 20% per layer down to 800 km. The mesh comprised 157 (N–S) x 223 (E–W) × 114 (vertical) cells, ~ 3.99 million cells in total, and the padding was rotated by 30° westward. The starting resistivity model assigned 100 Ohm.m from the surface to 400 km depth, 10 Ohm.m between 400 and 670 km, and 1 Ohm.m below 670 km based on mantle resistivity^48^. Seawater with resistivity 0.3 Ohm.m was included in the model.

Sixteen periods (10–10,000 s; five per decade) were inverted. Error floors of 5% were applied to each impedance component and 0.02 to the vertical field. Site-by-site distortion parameters were included but heavily penalized so that structure is expressed primarily in the resistivity model. Regularization had been tested in the range (0.1–1) both horizontal and vertical smoothing, acknowledging ~ 55 km inter-site spacing and stronger shallow vertical gradients. Additional smoothing was enforced in the upper 1,000 m to encourage laterally consistent near-surface resistivities compatible with regional sedimentary continuity.

The final model obtained with horizontal and vertical smoothing parameters of (tauH = tauV = 1) achieved an overall root-mean-square (RMS) misfit of 1.92 and is adopted as the preferred model. Fits between observed and modelled responses are shown in the Supplementary Information. Phase invariant and normalised phase invariant responses for periods of 21 s, 215 s, and 2154 s are presented in Supplementary Figure S2. Apparent resistivity invariant and normalised apparent resistivity invariant fits for the same periods are shown in Supplementary Figure S3. Fits to the real components of the induction arrows (Tx and Ty), together with their normalised equivalents, are displayed in Supplementary Figures S4 and S5 for periods of 21 s, 215 s, and 2154 s.

Thermal–electrical structure curves for Fig. 7 were generated in MATE^35^ assuming a lherzolite composition and three water contents (0, 100, 200 ppm). Moho depths of 35 km (volcanic, Monaro) and 45 km (non-volcanic, Orroroo) were imposed, with a maximum depth of 200 km. The geotherm was taken from the Geoscience Australia dataset^33^ (https://ecat.ga.gov.au/geonetwork/srv/eng/catalog.search#/metadata/149411). Water partitioning was implemented for pyroxene/olivine^49^ and garnet/olivine^50^, and resistivity followed mineral-specific models for olivine^51^ and pyroxene^52,53^.

## Supplementary Information

Below is the link to the electronic supplementary material.

Supplementary Material 1

## Data Availability

All magnetotelluric data are available from the Geoscience Australia (http://dx.doi.org/10.11636/Record.2018.021), the State Government of South Australia SARIG (https://map.sarig.sa.gov.au/) and National Computational Infrastructure (NCI) (https://www.nci.org.au/). Elevation, magnetic and gravity data were sourced from Geoscience Australia’s Geophysical Archive Data Delivery System (https://portal.ga.gov.au/persona/gadds) released under a CC BY 4.0 license (https://www.ga.gov.au/copyright). Depth to Proterozoic basement were obtained from Geognostics OZ SEEBASE 2021 (https://www.geognostics.com/oz-seebase-2021). Sedimentary basin data were sourced from the Geoscience Australia Portal (https://portal.ga.gov.au/) and are copyright Commonwealth of Australia (Geoscience Australia) 2018, released under the Creative Commons Attribution 4.0 International License (CC BY 4.0). Magnetotelluric numerical modelling was undertaken using using Viridien Geotools V.4.0.3.12574 (https://www.viridiengroup.com/expertise/multiphysics-imaging/geotools).

## Code availability

The custom scripts used in this study for data processing and visualization are available from the corresponding author upon reasonable request.

## References

[1] Glen, R. A. The Tasmanides of Eastern Australia. *Geol. Soc. Lond. Spec. Publ.* **246**, 23–96. 10.1144/GSL.SP.2005.246.01.02 (2005).

[2] Rosenbaum, G. The Tasmanides: Phanerozoic tectonic evolution of Eastern Australia. *Annu. Rev. Earth Planet. Sci.* **46**, 291–325. 10.1146/annurev-earth-082517-010146 (2018).

[3] Buckman, S. et al. The Watonga Formation and Tacking Point Gabbro, Port Macquarie, Australia: Insights into crustal growth mechanisms on the eastern margin of Gondwana. *Gondwana Res.* **28**, 133–151. 10.1016/j.gr.2014.02.013 (2015).

[4] Collins, W. J. Nature of extensional accretionary orogens. *Tectonics* **21**, 6-1–6-12. 10.1029/2000TC001272 (2002).

[5] Collins, W. J. Hot orogens, tectonic switching, and creation of continental crust. *Geology* **30**, 535–538. 10.1130/0091-7613(2002)030 (2002).

[6] Cawood, P. A. Terra Australis Orogen: Rodinia breakup and development of the Pacific and Iapetus margins of Gondwana during the Neoproterozoic and Paleozoic. *Earth-Sci. Rev.* **69**, 249–279. 10.1016/j.earscirev.2004.09.001 (2005).

[7] Korsch, R. J. & Doublier, M. P. Major crustal boundaries of Australia, and their significance in mineral systems targeting. *Ore Geol. Rev.* **76**, 211–228. 10.1016/j.oregeorev.2015.05.010 (2016).

[8] Vasconcelos, P. M., Knesel, K. M., Cohen, B. E. & Heim, J. A. Geochronology of the Australian Cenozoic: a history of tectonic and igneous activity, weathering, erosion, and sedimentation. *Aust. J. Earth Sci.* **55**, 865–914. 10.1080/08120090802120120 (2008).

[9] Davies, D. R. & Rawlinson, N. On the origin of recent intraplate volcanism in Australia. *Geology* **42**, 1031–1034. 10.1130/g36093.1 (2014).

[10] Ball, P. W., Czarnota, K., White, N. J., Klöcking, M. & Davies, D. R. Thermal structure of Eastern Australia’s upper mantle and its relationship to Cenozoic volcanic activity and dynamic topography. *Geochem. Geophys. Geosyst.*10.1029/2021GC009717 (2021).

[11] Mather, B. R. et al. Intraplate volcanism triggered by bursts in slab flux. *Sci. Adv.* **6**, 953–969. 10.1126/sciadv.abd0953 (2020). 10.1126/sciadv.abd0953 PMC7744089 33328233

[12] King, S. D. & Anderson, D. L. Edge-driven convection. *Earth Planet. Sci. Lett.* **160**, 289–296. 10.1016/S0012-821X(98)00089-2 (1998).

[13] Conrad, C. P., Bianco, T. A., Smith, E. I. & Wessel, P. Patterns of intraplate volcanism controlled by asthenospheric shear. *Nat. Geosci.* **4**, 317–321. 10.1038/ngeo1111 (2011).

[14] Aivazpourporgou, S., Thiel, S., Hayman, P. C., Moresi, L. N. & Heinson, G. Decompression melting driving intraplate volcanism in Australia: Evidence from magnetotelluric sounding. *Geophys. Res. Lett.* **42**, 346–354. 10.1002/2014GL060088 (2015).

[15] Heinson, G. et al. Lower crustal resistivity signature of an orogenic gold system. *Sci. Rep.* **11**, 15807. 10.1038/s41598-021-94531-8 (2021). 34349155 10.1038/s41598-021-94531-8 PMC8338967

[16] Kay, B., Heinson, G. & Boren, G. Multiscale resistivity mapping from an intracontinental hydrothermal mineral system, Adelaide Rift Complex, Australia. *Gondwana Res.* **129**, 292–304. 10.1016/j.gr.2023.12.012 (2024).

[17] Kay, B. et al. Lithospheric architecture of the Curnamona Province, Australia. *Gondwana Res.* **144**, 49–63. 10.1016/j.gr.2025.04.001 (2025).

[18] Kirkby, A. L. et al. Lithospheric architecture of a Phanerozoic orogen from magnetotellurics: AusLAMP in the Tasmanides, Southeast Australia. *Tectonophysics* **793**, 228560. 10.1016/j.tecto.2020.228560 (2020).

[19] Manassero, M. C. et al. Lithospheric structure and melting processes in Southeast Australia: New constraints from joint probabilistic inversions of 3D magnetotelluric and seismic data. *J. Geophys. Res. Solid Earth* **129**, e2023JB028257. 10.1029/2023JB028257 (2024).

[20] Ostersen, T. *Geoelectric structure of the Tasmanian lithosphere from multi-scale magnetotelluric data* PhD thesis, University of Tasmania, (2023).

[21] Robertson, K., Heinson, G. & Thiel, S. Lithospheric reworking at the Proterozoic–Phanerozoic transition of Australia imaged using AusLAMP magnetotelluric data. *Earth Planet. Sci. Lett.* **452**, 27–35. 10.1016/j.epsl.2016.07.036 (2016).

[22] Jennings, S., Heinson, G., Hasterok, D. & Kay, B. Magnetotelluric support for edge-driven convection and shear-driven upwelling in the Newer Volcanics Province. *Sci. Rep.* **13**, 5543. 10.1038/s41598-023-32403-z (2023). 37016012 10.1038/s41598-023-32403-z PMC10073071

[23] Chamalaun, F. H. Geomagnetic deep sounding experiment in the central Flinders Ranges of South Australia. *Phys. Earth Planet. Inter.* **37**, 174–182. 10.1016/0031-9201(85)90050-0 (1985).

[24] Kay, B., Heinson, G. & Brand, K. Crustal magnetotelluric imaging of a Paleoproterozoic graphitic suture zone, Curnamona Province, Australia. *Gondwana Res.* **106**, 1–14. 10.1016/j.gr.2021.12.005 (2022).

[25] Parkinson, W. D. & Hermanto, R. The Tamar conductivity anomaly. *Explor. Geophys.* **17**, 34–35. 10.1071/EG986034 (1986).

[26] Parkinson, W. D. et al. The Tamar conductivity anomaly. *Phys. Earth Planet. Inter.* **52**, 8–22. 10.1016/0031-9201(88)90053-2 (1988).

[27] Mackie, R. L. & Madden, T. R. Three-dimensional magnetotelluric inversion using conjugate gradients. *Geophys. J. Int.* **115**, 215–229. 10.1111/j.1365-246X.1993.tb05600.x (1993).

[28] Yang, X. Origin of high electrical conductivity in the lower continental crust: A review. *Surv. Geophys.* **32**, 875–903. 10.1007/s10712-011-9145-z (2011).

[29] Kennett, B. L. N. et al. Refining the Moho across the Australian continent. *Geophys. J. Int.* **233**, 1863–1877. 10.1093/gji/ggad035 (2023).

[30] Hoggard, M. J. et al. Global distribution of sediment-hosted metals controlled by craton edge stability. *Nat. Geosci.* **13**, 504–510. 10.1038/s41561-020-0593-2 (2020).

[31] Kennett, B. L. N., Fichtner, A., Fishwick, S. & Yoshizawa, K. Australian Seismological Reference Model (AuSREM); mantle component. *Geophys. J. Int.* **192**, 871–887. 10.1093/gji/ggs065 (2013).

[32] Fishwick, S. & Rawlinson, N. 3-D structure of the Australian lithosphere from evolving seismic datasets. *Aust. J. Earth Sci.* **59**, 809–826. 10.1080/08120099.2012.702319 (2012).

[33] Hoggard, M. et al. Thermomechanical models of the Australian plate. *Geoscience Australia*. 10.26186/149411 (2024).

[34] Direen, N. G. & Crawford, A. J. The Tasman Line: Where is it, what is it, and is it Australia’s Rodinian breakup boundary?. *Aust. J. Earth Sci.* **50**, 491–502. 10.1046/j.1440-0952.2003.01005.x (2003).

[35] Özaydın, S. & Selway, K. MATE: An analysis tool for the interpretation of magnetotelluric models of the mantle. *Geochem. Geophys. Geosyst.* **21**, e2020GC009126. 10.1029/2020GC009126 (2020).

[36] Rudnick, R. L. & Fountain, D. M. Nature and composition of the continental crust: A lower crustal perspective. *Rev. Geophys.* **33**, 267–309. 10.1029/95RG01302 (1995).

[37] Yardley, B. W. D. & Valley, J. W. The petrologic case for a dry lower crust. *J. Geophys. Res. Solid Earth* **102**, 12173–12185. 10.1029/97JB00508 (1997).

[38] Yang, X. Orientation-related electrical conductivity of hydrous olivine, clinopyroxene and plagioclase and implications for the structure of the lower continental crust and uppermost mantle. *Earth Planet. Sci. Lett.* **317–318**, 241–250. 10.1016/j.epsl.2011.11.011 (2012).

[39] Yang, X., Keppler, H., McCammon, C. & Ni, H. Electrical conductivity of orthopyroxene and plagioclase in the lower crust. *Contrib Mineral Petr* **163**, 33–48. 10.1007/s00410-011-0657-9 (2012).

[40] Yang, X. et al. Effect of water on the electrical conductivity of lower crustal clinopyroxene. *J. Geophys. Res.*10.1029/2010JB008010 (2011).

[41] Chave, A. D. & Thomson, D. J. Bounded influence magnetotelluric response function estimation. *Geophys. J. Int.* **157**, 988–1006. 10.1111/j.1365-246X.2004.02203.x (2004).

[42] Chave, A. D. & Thomson, D. J. A bounded influence regression estimator based on the statistics of the hat matrix. *J. R. Stat. Soc. C Appl. Stat.* **52**, 307–322. 10.1111/1467-9876.00406 (2003).

[43] Krieger, L. & Peacock, J. R. MTpy: A Python toolbox for magnetotellurics. *Comput. Geosci.* **72**, 167–175. 10.1016/j.cageo.2014.07.013 (2014).

[44] Booker, J. R. The magnetotelluric phase tensor: A critical review. *Surv. Geophys.* **35**, 7–40. 10.1007/s10712-013-9234-2 (2014).

[45] Caldwell, T. G., Bibby, H. M. & Brown, C. The magnetotelluric phase tensor. *Geophys. J. Int.* **158**, 457–469. 10.1111/j.1365-246X.2004.02281.x (2004).

[46] Rodi, W. & Mackie, R. L. Nonlinear conjugate gradients algorithm for 2-D magnetotelluric inversion. *Geophysics* **66**, 174–187. 10.1190/1.1444893 (2001).

[47] Mackie, R. L. & Madden, T. R. 3-Dimensional magnetotelluric inversion using conjugate gradients. *Geophys. J. Int.* **115**, 215–229. 10.1111/j.1365-246X.1993.tb05600.x (1993).

[48] Grayver, A. V., van Driel, M. & Kuvshinov, A. V. Three-dimensional magnetotelluric modelling in spherical Earth. *Geophys. J. Int.* **217**, 532–557. 10.1093/gji/ggz030 (2019).

[49] Demouchy, S., Shcheka, S., Denis, C. M. M. & Thoraval, C. Subsolidus hydrogen partitioning between nominally anhydrous minerals in garnet-bearing peridotite. *Am. Mineral.* **102**, 1822–1831. 10.2138/am-2017-6089 (2017).

[50] Novella, D. et al. The distribution of H2O between silicate melt and nominally anhydrous peridotite and the onset of hydrous melting in the deep upper mantle. *Earth Planet. Sci. Lett.* **400**, 1–13. 10.1016/j.epsl.2014.05.006 (2014).

[51] Dai, L. & Karato, S.-i. High and highly anisotropic electrical conductivity of the asthenosphere due to hydrogen diffusion in olivine. *Earth Planet. Sci. Lett.* **408**, 79–86. 10.1016/j.epsl.2014.10.003 (2014).

[52] Dai, L. & Karato, S.-i. Electrical conductivity of pyrope-rich garnet at high temperature and high pressure. *Phys. Earth Planet. Inter.* **176**, 83–88. 10.1016/j.pepi.2009.04.002 (2009).

[53] Liu, H., Zhu, Q. & Yang, X. Electrical conductivity of OH-bearing omphacite and garnet in eclogite: The quantitative dependence on water content. *Contrib. Mineral. Petrol.* **174**, 1–15. 10.1007/s00410-019-1593-3 (2019).

