# Supplementary material for: Mantle melting and lithospheric structure beneath eastern Australia’s Cenozoic volcanoes from 3D magnetotellurics
Source: Sci Rep. 2026 Mar 19;16:14214. doi: 10.1038/s41598-026-44483-8 (PMC13139488; doi:10.1038/s41598-026-44483-8)
Supplement: Supplementary file 1 — Supplementary Material 1 [file 41598_2026_44483_MOESM1_ESM.docx]

**Mantle Melting and Lithospheric Structure Beneath Eastern Australia’s Cenozoic Volcanoes from 3D Magnetotellurics**

**Supplementary Section**

Relly Margiono^a,b^* and Graham Heinson^a^

^a^Department of Earth Sciences, Adelaide University, Adelaide SA 5005, Australia

^b^Indonesia State College of Meteorology, Climatology, and Geophysics, Tangerang, Indonesia

[*relly.margiono@adelaide.edu.au](mailto:*relly.margiono@adelaide.edu.au)

Version 25^th^ February 2026

**Phase Tensor**

The phase tensor method provides a robust framework for assessing subsurface dimensionality and large-scale resistivity variations while remaining insensitive to galvanic distortion in electric field measurement^1,2^. Figure S1 presents phase tensor ellipses coloured by the invariant phase for periods of 21 s, 215 s, and 2,154 s, corresponding to progressively increasing depths of investigation. In broad terms, red coloured ellipses (> 45°) indicate that the earth is becoming more conductive with depth, and blue coloured ellipses (< 45°) indicate that the earth is becoming more resistive with depth.

At the shortest period (21 s), ellipses display pronounced spatial variability in both orientation and colour, consistent with heterogeneous crustal resistivity structures. At this period, elevated phase values are observed in the north-western Tasmanides (Curnamona Province), the eastern Tasmanides, and Tasmania, indicating a relatively low resistive crust. In contrast, the Thomson Orogen and the northern Delamerian Orogen are characterised by lower phase values (<45°), suggesting a more resistive crustal regime. At intermediate periods (215 s), the phase tensor ellipses become more spatially coherent across large regions, indicating the development of laterally continuous lithospheric resistivity patterns. At the longest period (2,154 s), ellipses are rounder and relatively uniform in colour, implying that the upper mantle exhibits relatively homogeneous electrical properties and limited lateral variation at these depths.


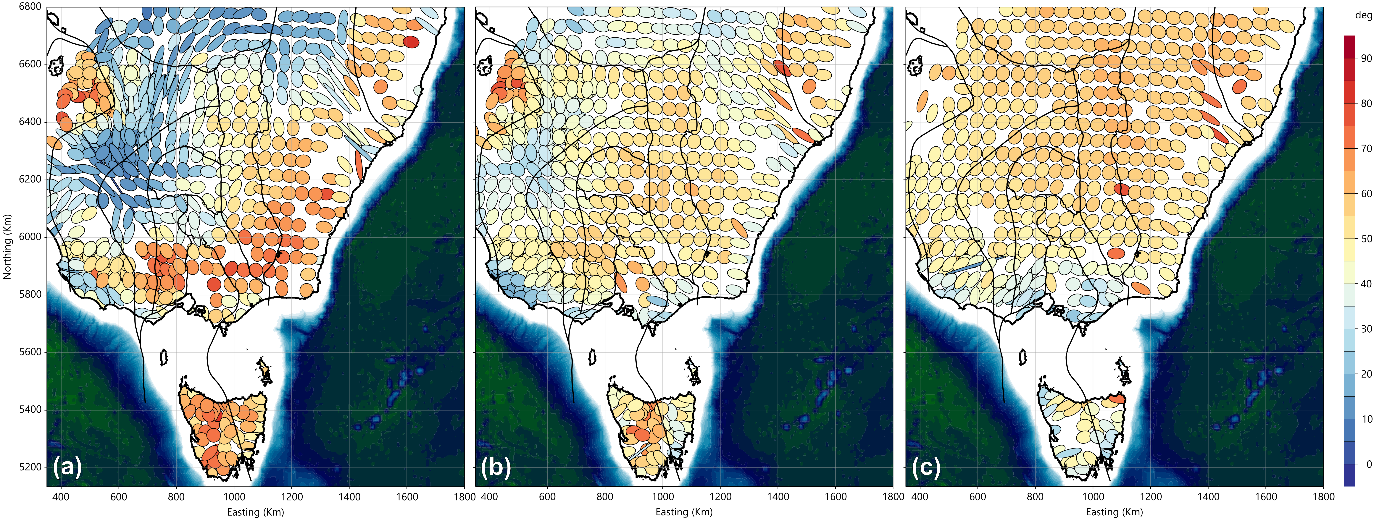


Figure S1: Phase tensor maps for periods of (a) 21 s; (b) 215 s; (c) 2,154 , coloured by the invariant phase. Areas are shown in UTM 54S projection.

**Model Fits**

Details of the preferred three-dimensional model are provided in the Methods section of the main paper. The optimal regularisation parameters were tauH = 1 and tauV = 1. Using these parameters, the inversion incorporates the full dataset of 803 MT and GDS sites, comprising 530 long-period MT (5 component) responses (10–10,000 s), 203 broadband MT responses (modelled in the bandwidth 10-1,000 s), and 70 solely GDS responses (1,000-10,000 s), distributed over ~1,550 × 1,450 km, and achieves an overall root-mean-square (RMS) misfit of 1.92. Although the inversion simultaneously fits all components of the impedance tensor, presenting fits to individual tensor elements is less informative. Instead, model performance is more effectively evaluated using the impedance invariant, defined here as the determinant of the impedance tensor. This complex invariant is expressed in terms of both apparent resistivity and phase, shown as observed data in the left-hand panels, and as misfits in the right-hand panels. The phase misfits are in degrees and are the difference between the observed and modelled values in the range of ±20°. Apparent resistivity misfits are shown as the ratio of observed divided by modelled values in the range of 2 to 0.5. Additionally, fits to the real components of the induction arrows (Tx and Ty), together with the difference between the observed and modelled values in the range of ±0.2, are presented to indicate the consistency of the anomalous vertical magnetic field components. The inversion incorporates error floors of 5% on the impedance components, corresponding to uncertainties of approximately 10% in apparent resistivity and ~2.9° in phase. Given these error assumptions and the achieved RMS misfit of 1.92, the expected data fits should fall within colour ranges immediately above and below a ratio of 1 (green to yellow) for the normalised invariant apparent resistivity, and within colour ranges immediately above and below zero difference (green to yellow) for the normalised invariant phase.

Observed and normalised invariant are shown in Figure S2. At a period of 21 s, elevated invariant phase values are observed across the eastern Tasmanides, Tasmania, and the Curnamona Province, whereas lower phase values characterise the Thomson Orogen and the northern Delamerian Orogen. At an intermediate period of 215 s, the Curnamona Province continues to exhibit relatively high phase values. By the longest period considered (2,154 s), the invariant phase distribution becomes more spatially uniform, indicating increasingly homogeneous electrical properties at greater depths. The phase misfit maps show generally a good fit of <5°, with no obvious spatial correlation across multiple sites. Out of >800 sites, only a few (<10) have misfits that are higher than 20°. These misfits are likely due to localised resistivity heterogeneity and/or topography and bathymetry that cannot be captured with a grid cell of 10 km. We have included these sites in the inversion as they are not deemed to bias the final model in any significant manner.


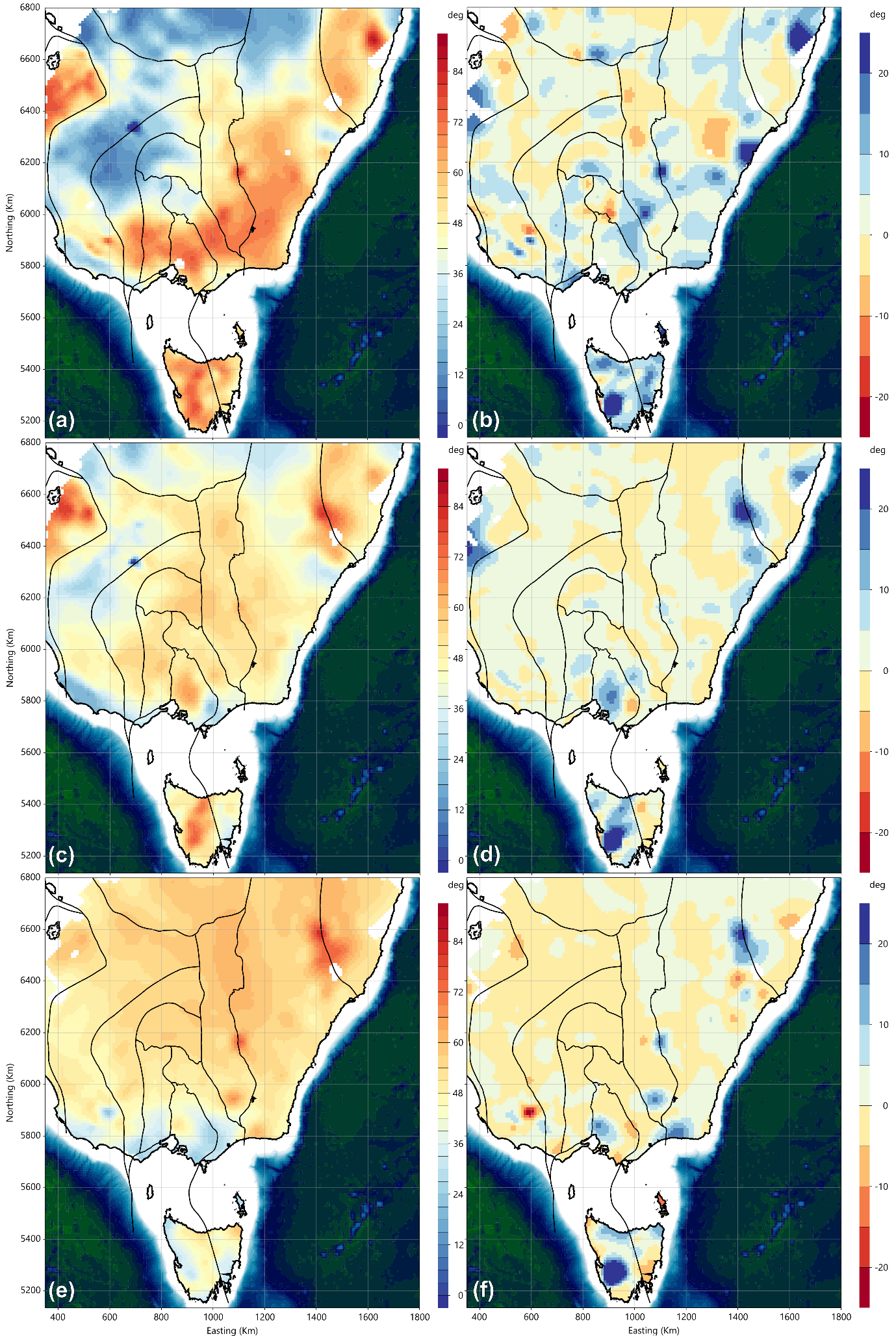


Figure S2: Phase invariant for periods of (a) 21 s; (c) 215 s; (e) 2,154 s. Normalised phase invariant (observed – modelled) for periods of (b) 21 s; (d) 215 s; (f) 2,154 s. Areas are shown in UTM 54S projection.

Observed and normalised invariant apparent resistivity are shown in Figure S3. At a period of 21 s, the apparent resistivity distribution across the Tasmanides is highly heterogeneous, reflecting strong lateral variations in crustal resistivity. At an intermediate period of 215 s, low apparent resistivity values are evident in the Curnamona Province, Tasmania, and along the southern margin of the New England Orogen. These conductive features persist at the longest period (2,154 s). The normalised apparent resistivity represents the ratio between observed and modelled responses, with a value of 2 indicating that the observed apparent resistivity is twice the modelled value, whereas 0.5 indicates the modelled response is twice the observed. A value of 1 signifies agreement between observed and modelled data. Across all three periods, the majority of normalised apparent resistivity values cluster near unity, indicating an overall good fit between the model and observations, with only limited areas exhibiting larger misfits that are not spatially correlated. As for the normalised phase, the localised misfits are most probably due to limitations of modelling topography, bathymetry and small-scale resistivity heterogeneities with a 10 km grid cell size.

Figures S4 and S5 present the real components of the induction arrows in the Tx and Ty directions, respectively, together with their normalised misfits (observed minus modelled) for periods of 21 s, 215 s, and 2,154 s. For both components, the observed real induction arrow component responses in x and y show coherent regional-scale patterns that are consistently reproduced by the 3D resistivity model across all periods. The normalised misfits are largely centred around zero, indicated by green to yellow colour ranges, in agreement with the applied vertical field error floor of 0.02 and demonstrating an overall good fit between observed and predicted responses. Misfits are spatially sparse and lack systematic regional correlation, suggesting that they primarily reflect local noise or small-scale resistivity variations at individual sites rather than deficiencies in the regional resistivity structure. The consistent performance of both Tx and Ty components across increasing periods indicates that the model adequately captures the depth-dependent induction response from shallow crustal levels to lithospheric depths.


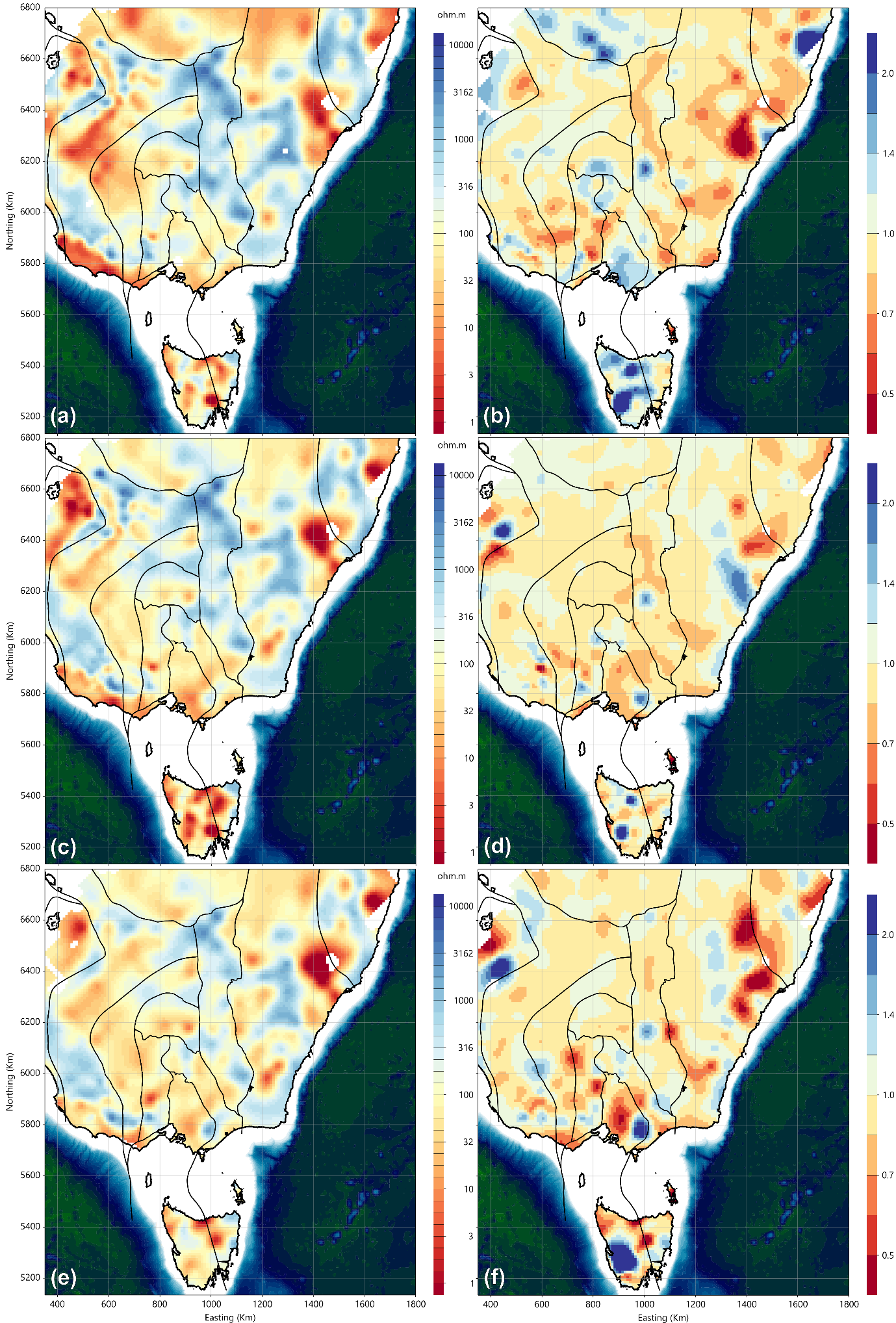


Figure S3: Invariant apparent resistivity for periods of (a) 21 s; (c) 215 s; (e) 2,154 s. Normalised invariant apparent resistivity (observed/modelled) for periods of (b) 21 s; (d) 215 s; (f) 2,154 s. Areas are shown in UTM 54S projection.


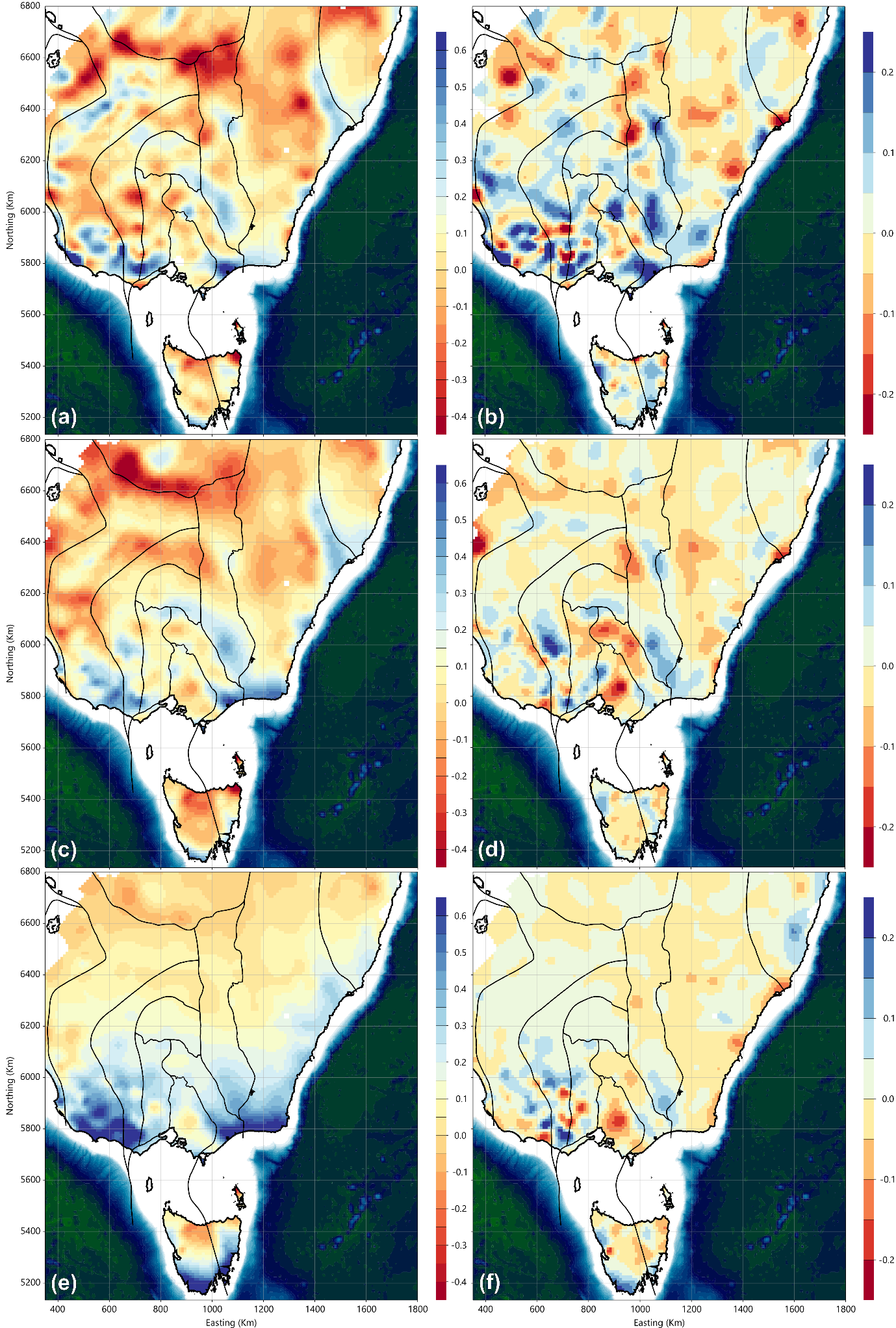


Figure S4: Real part of the induction arrow Tx for periods of (a) 21 s; (c) 215 s; (e) 2,154 s. Normalised real part of the induction arrow Tx (observed – modelled) for periods of (b) 21 s; (d) 215 s; (f) 2,154 s. Areas are shown in UTM 54S projection.


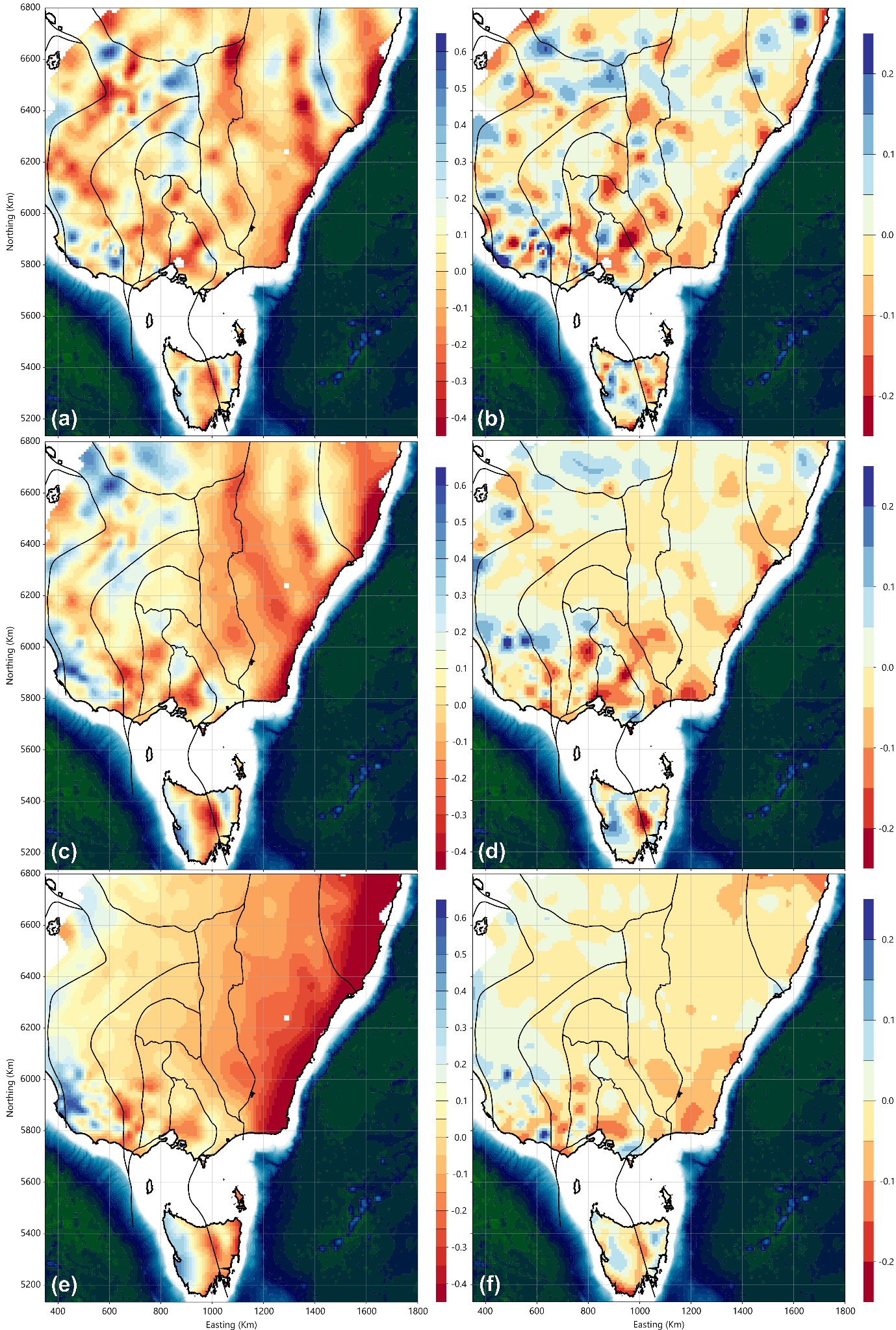


Figure S5: Real part of the induction arrow Ty for periods of (a) 21 s; (c) 215 s; (e) 2,154 s. Normalised real part of the induction arrow Ty (observed – modelled) for periods of (b) 21 s; (d) 215 s; (f) 2,154 s.

**Mantle Composition Modelling**

A lherzolite composition was chosen as a conservative option given few xenolith constraints for most of the Tasmanides. Partial melting of the mantle would typically lead to depletion to harzburgite (73% olivine, 25% orthopyroxene, 2% clinopyroxene) and alternatively metasomatism may enhance clinopyroxene content to wehrlite (71% olivine, 2% orthopyroxene, 27% clinopyroxene). We show in Figure S6 the MATE predicted mantle resistivities for the Monaro temperature profile for these three compositions with no hydration (0 ppm H_2_0), along with model estimates and standard deviations for volcano nodes of the 3D model. These compositional curves differ by about 0.2 log units in the depth range 100-200 km, and all are consistent with the modelled values.


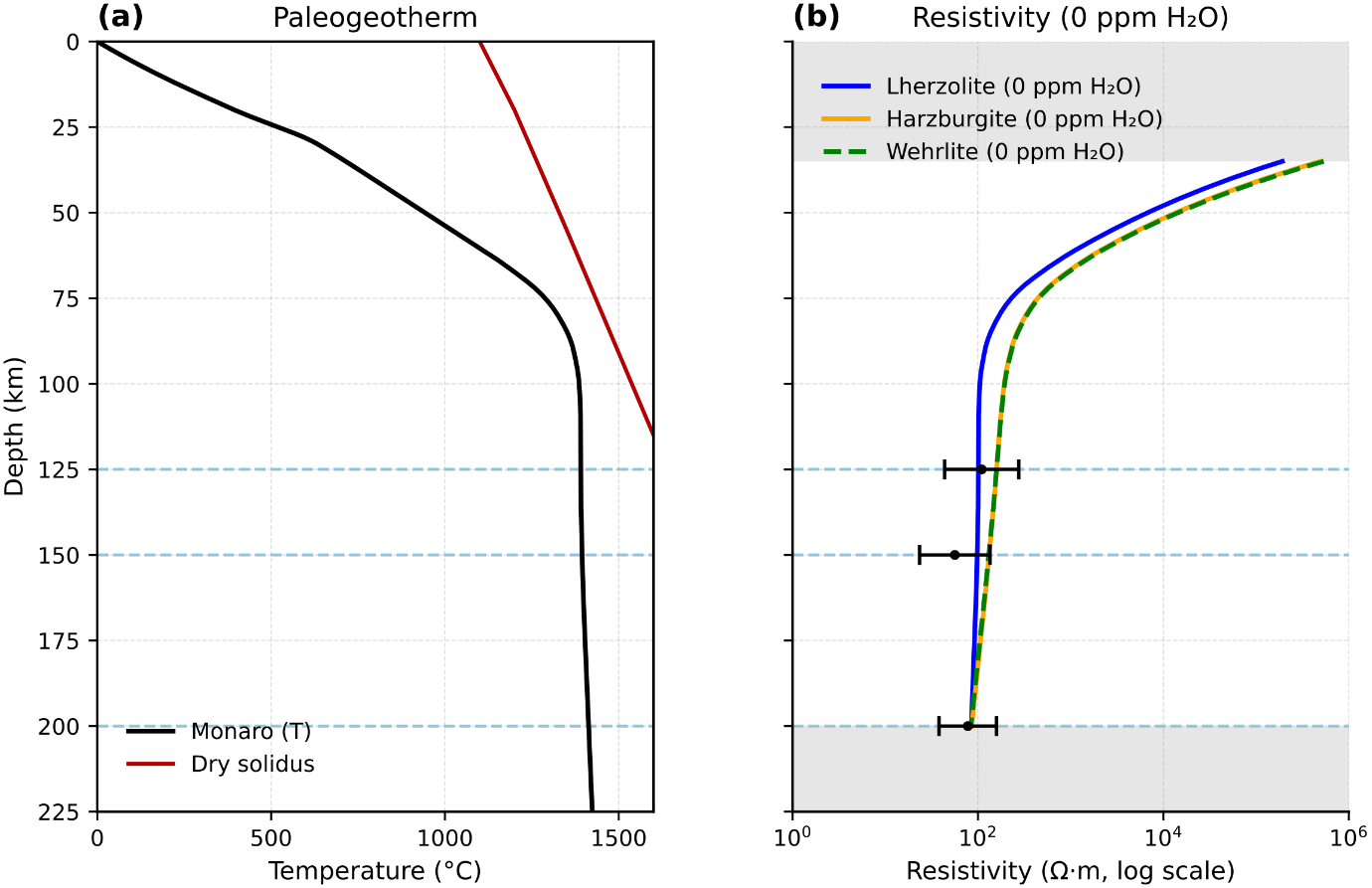


Figure S6: (a) Palaeogeotherm for Monaro (volcanic areas) and (b) predicted mantle resistivity profiles derived with MATE^3^ for dry lherzolite-harzburgite-wehrlite. Data with error bars are extracted from volcanic nodes in the 3D inversion.

**References**

1 Booker, J. R. The Magnetotelluric Phase Tensor: A Critical Review. *Surveys in Geophysics* **35**, 7-40, doi:10.1007/s10712-013-9234-2 (2014).

2 Caldwell, T. G., Bibby, H. M. & Brown, C. The magnetotelluric phase tensor. *Geophysical Journal International* **158**, 457-469, doi:10.1111/j.1365-246X.2004.02281.x (2004).

3 Özaydın, S. & Selway, K. MATE: An Analysis Tool for the Interpretation of Magnetotelluric Models of the Mantle. *Geochemistry, Geophysics, Geosystems* **21**, e2020GC009126, doi:<https://doi.org/10.1029/2020GC009126> (2020).
